# Multi-analytical characterization and radiation shielding assessment of natural ophiolitic rocks: a combined experimental, mineralogical, and simulation analysis

**DOI:** 10.1038/s41598-026-66379-3

**Published:** 2026-08-22

**Authors:** Mohamed A. Agha, Mohamed A. Abouelnour, Islam N. Fathy, Alaa A. Mahmoud, Hussein Oraby, Amir Ezzat, Maged E. Elfakharany, Islam M. Nabil, Abdelhalim S. Mahmoud

**Affiliations:** 1 Geology Department, Faculty of Science, Fayoum University, Fayoum, Egypt; 2 Civil Engineering Department, Faculty of Engineering, Fayoum University, Fayoum, Egypt; 3 Construction and Building Engineering Department, October High Institute for Engineering & Technology, Giza, Egypt; 4 Department of Chemical Engineering, Military Technical College, Cairo, Egypt; 5 Chemistry Department, Faculty of Science, Ain Shams University, Cairo, Egypt; 6 Raw Materials Institute, Housing and Building National Research Center (HBRC), Giza, Egypt; 7 Physics Department, Faculty of Science, Fayoum University, Fayoum, Egypt

**Keywords:** Ophiolites, Metavolcanics, Ophiolitic metasediment, Serpentinized peridotites, Metagabbro-diorite, Radiation shielding, Environmental sciences, Materials science, Solid Earth sciences

## Abstract

Ophiolitic suites derived from oceanic crust are rich in ferromagnesian and mafic minerals and exhibit relatively high densities, making them promising and economical materials for radiation shielding. This study evaluates ophiolitic rocks exposed along the Marsa Alam–Idfu road in the southern Eastern Desert of Egypt as natural radiation shielding materials, including mafic metavolcanics, mafic schist, serpentinite peridotites, and hornblende metagabbro-diorite. Comprehensive geochemical analyses were conducted using polarizing microscopy, X-ray diffraction (XRD), thermogravimetric analysis (TGA), and Fourier-transform infrared spectroscopy (FTIR) to constrain the mineralogical frameworks. Experimental γ-attenuation measurements were performed using a P-HPGe detector for Cs-137 (0.662 MeV) and Co-60 (1.173 and 1.332 MeV) sources. Monte Carlo simulations (MCNP-5) and EpiXs software were also employed to model photon interactions and validate the experimental results. The mineralogical assemblages of the investigated samples which predominantly composed of serpentine, chlorite, amphibole, plagioclase, talc, and carbonates, play a key role in controlling their radiation shielding properties. Geochemically, the rocks display a major oxide compositional range from ultramafic to mafic. Serpentinized peridotites, in particular, exhibit high MgO content and low silica, while mafic metavolcanics and schists are rich in Al_2_O_3_ and FeO. Radiation shielding results showed that the linear attenuation coefficients (µ) exhibited a pronounced decline with increasing photon energy, controlled by photoelectric and Compton interactions, with serpentinized peridotites showing the highest attenuation due to its greater density and high iron oxide content. Simulated and experimentally calculated µ values showed strong agreement (φ = 2.431%), while experimental results were slightly higher, with deviations ranging from − 3.6% to − 9.2%. The obtained trends of attenuation coefficient, half-value layer, tenth-value layer, effective atomic number, and radiation protection efficiency (RP_eff_) further confirmed the energy-dependent attenuation behavior, identifying serpentinized peridotites as the most efficient γ-ray shielding material among the investigated rocks. Furthermore, Neutron-shielding assessment revealed that serpentinized peridotites exhibited the highest fast neutron removal cross-Sect.  (0.097 cm^− 1^) and the lowest half-value layer (7.121 cm) and relaxation length (10.273 cm), indicating superior neutron attenuation performance among the investigated lithologies.

## Introduction

The proliferation of artificial ionizing radiation–generating devices, including X-ray and γ-ray systems, has fundamentally transformed numerous sectors encompassing industrial processes, medical diagnostics and therapeutics, and nuclear energy applications. Consequently, the development of advanced radiation shielding materials and the accurate evaluation of their shielding performance are essential for ensuring protection against the harmful effects of ionizing radiation and maintaining a safe living environment^1,2^. This widespread adoption is a direct result of significant technological strides that have enhanced the precision, efficiency, and accessibility of radiation-based systems^3,4^. However, despite these transformative benefits, the potential health hazards associated with ionizing radiation remain a critical concern, necessitating robust evaluation and mitigation strategies. The biological implications of such exposure are well-documented, spanning a spectrum from chronic long-term effects to acute syndromes. Prolonged exposure to high-energy photons can induce cellular damage and genomic instability, potentially leading to carcinogenesis. Conversely, acute exposure can manifest as immediate physiological distress including gastrointestinal symptoms such as nausea and vomiting which, in extreme cases, may prove fatal^5^. The fundamental mechanism underlying these adverse effects involves the interaction of high-energy photons with human tissue, resulting in the ionization of water molecules within cellular structures. This ionization process poses a significant threat to genetic material, as it induces damage to both the external surface and internal components of DNA molecules, potentially compromising cellular integrity and function^5,6^. The environmental and biological hazards posed by various forms of ionizing radiation, including neutrons, γ-rays, and X-rays, extend beyond human health concerns to encompass broader ecological implications affecting both terrestrial and aquatic ecosystems. These radiological threats necessitate the implementation of comprehensive protection strategies that prioritize the development and deployment of effective radiation attenuation and shielding materials^7,8^. Consequently, the scientific community has demonstrated sustained interest in identifying and characterizing superior materials capable of providing enhanced radiation protection across diverse applications and energy spectra.

The fundamental principle underlying radiation protection strategies involves the strategic placement of shielding and attenuation materials as protective barriers between radiation sources and potentially exposed individuals or environments. These materials function by absorbing or deflecting incident radiation, thereby reducing the intensity of harmful radiation that reaches protected areas and minimizing occupational and public exposure risks^9^. The selection of appropriate shielding materials represents one of the cornerstone concepts in radiation protection protocols, as the effectiveness of protection systems is intrinsically linked to the physical and chemical properties of the constituent materials employed. Natural ores, being composed of diverse elements and mineral compounds, provide considerable benefits for neutron and gamma radiation shielding due to their superior attenuation efficiency, wide-range shielding performance, and high resistance to radiation-related deterioration^10^. Heavy and dense materials, including metallic substances such as steel and lead, as well as composite materials like polyethylene-based composites and concrete formulations, have demonstrated proven efficacy in attenuating radiation across broad energy ranges^11–14^. The effectiveness of these materials is primarily attributed to their high atomic numbers and densities, which facilitate increased interaction probabilities with incident photons and particles. The ongoing investigation into radiation shielding materials has expanded to include polymeric substances, natural geological materials, rubber-based composites, ceramic materials, brick compositions, and various metallic alloys, each offering unique advantages and limitations depending on the specific application requirements^15–22^.

Despite the extensive research conducted on conventional radiation shielding materials like concretes^23–25^, glasses^26–30^, and metallic alloys^20,31,32^, there remains significant potential for the exploration of naturally occurring geological materials that may offer cost-effective alternatives to traditional synthetic composites. Natural rocks, in particular, present compelling opportunities due to their inherent mineral compositions, variable densities, and potential abundance in certain geological settings. The utilization of natural materials for radiation shielding applications aligns with contemporary sustainability principles while potentially reducing material costs and environmental impacts associated with synthetic material production. Among the natural geological materials investigated for radiation shielding applications, various rock types have demonstrated promising attenuation performance. For instance, Obaid et al. (2018)^33^ evaluated the gamma-ray shielding characteristics of seven natural rocks, including basalt, granite, limestone, and marble varieties. The results revealed that pink marble exhibited the highest mass attenuation coefficient, effective atomic number, and electron density, together with the lowest half-value layer and buildup factors, indicating superior gamma-ray shielding efficiency. Bantan et al. (2020)^34^ assessed the gamma-ray shielding performance of several Egyptian natural rocks, including basalt, rhyolite, and limestone, through experimental measurements, Monte Carlo simulations, and theoretical calculations. The results demonstrated that basalt samples exhibited the highest attenuation capability, characterized by higher linear attenuation coefficients, lower HVL values, and lower buildup factors than the other investigated rocks, highlighting basalt as a promising natural material for radiation shielding applications. Libeesh et al. (2021)^35^ investigated the radiation shielding performance of mafic and ultramafic rocks, namely gabbro, peridotite, and pyroxenite, using Monte Carlo simulations and Phy-X calculations. Their findings demonstrated that gabbro exhibited the highest mass attenuation coefficient and effective atomic number, together with the lowest HVL values across the investigated energy range, indicating superior gamma-ray shielding capability compared with peridotite and pyroxenite. Yahsi Çelen et al. (2021)^36^ investigated the gamma- and neutron-shielding performance of several igneous rocks, including granite, granodiorite, and andesite. The results identified granite as the most effective shielding material, exhibiting the highest linear and mass attenuation coefficients, effective atomic number, and neutron removal cross-section, together with the lowest HVL, TVL, and mean free path values. The study further confirmed that radiation attenuation efficiency is strongly influenced by rock density and decreases with increasing photon energy. Al-Buriahi et al. (2021)^22^ evaluated the radiation shielding characteristics of several natural rocks, including olivine basalt, jet black granite, limestone, sandstone, and dolerite, using the EPICS2017 and XCOM databases. The results demonstrated that all investigated rocks exhibited superior shielding performance compared with conventional concrete and attenuation properties comparable to commercial radiation-shielding glasses. Among the studied samples, limestone and jet-black granite showed the highest effective atomic numbers, indicating enhanced photon attenuation capability. Günoğlu et al. (2024)^37^ experimentally and theoretically investigated the gamma-ray shielding performance of several igneous rocks from Turkey. The results showed that attenuation properties improved with increasing rock density and alkali-silica content, while the trachy-basalt sample exhibited the highest linear attenuation coefficient and radiation protection efficiency, together with the lowest HVL, TVL, and MFP values, indicating superior gamma-ray shielding effectiveness among the studied rocks. Abd El-Azeem and Harpy (2024)^38^ investigated the radiation attenuation characteristics of several natural rocks, including claystone, bentonitic claystone, bentonitic shale, sandstone, and basalt, through experimental measurements and XCOM-based calculations. The results demonstrated that basalt exhibited the most favorable shielding performance, characterized by higher attenuation coefficients and lower HVL, TVL, and MFP values than the other investigated rocks. Furthermore, increasing the basalt thickness significantly enhanced attenuation efficiency, reaching up to 77.1% for uranium-based radiation sources. Alshamari et al. (2026)^39^ evaluated the structural, physical, and radiation shielding properties of Lece volcanic rock and slag rock. The results revealed that Lece rock exhibited superior gamma- and neutron-shielding performance owing to its higher density and greater concentrations of Fe, Ca, Ti, and Si oxides, resulting in higher mass attenuation coefficients and neutron removal cross-sections than slag rock. The study also highlighted the potential of both rocks as sustainable and cost-effective materials for lightweight shielding systems and radiation-shielding aggregates. Kılıç and Yılmaz (2025)^40^ investigated the gamma- and neutron-shielding performance of serpentinite rocks from the Guleman ophiolite complex, with particular emphasis on the influence of rare earth elements (REEs). The results demonstrated that REE content, especially gadolinium, significantly enhanced thermal neutron attenuation, while crystalline water content improved fast-neutron shielding. Among the investigated samples, Sr-2 exhibited the best overall shielding performance for both gamma rays and neutrons, with an HVL approximately 58% lower than that of standard concrete. Azer et al. (2026)^41^ investigated the physico-mechanical and gamma-ray shielding properties of granodiorite from the Eastern Desert of Egypt. The results demonstrated that the studied granodiorite possesses excellent mechanical performance, including high compressive and flexural strengths, together with effective gamma-ray attenuation over the energy range of 0.015–15 MeV. Among the investigated samples, Gd32 exhibited the highest density, superior mechanical properties, and the best shielding performance, confirming the strong influence of density on radiation attenuation efficiency.

**Fig. 1:**
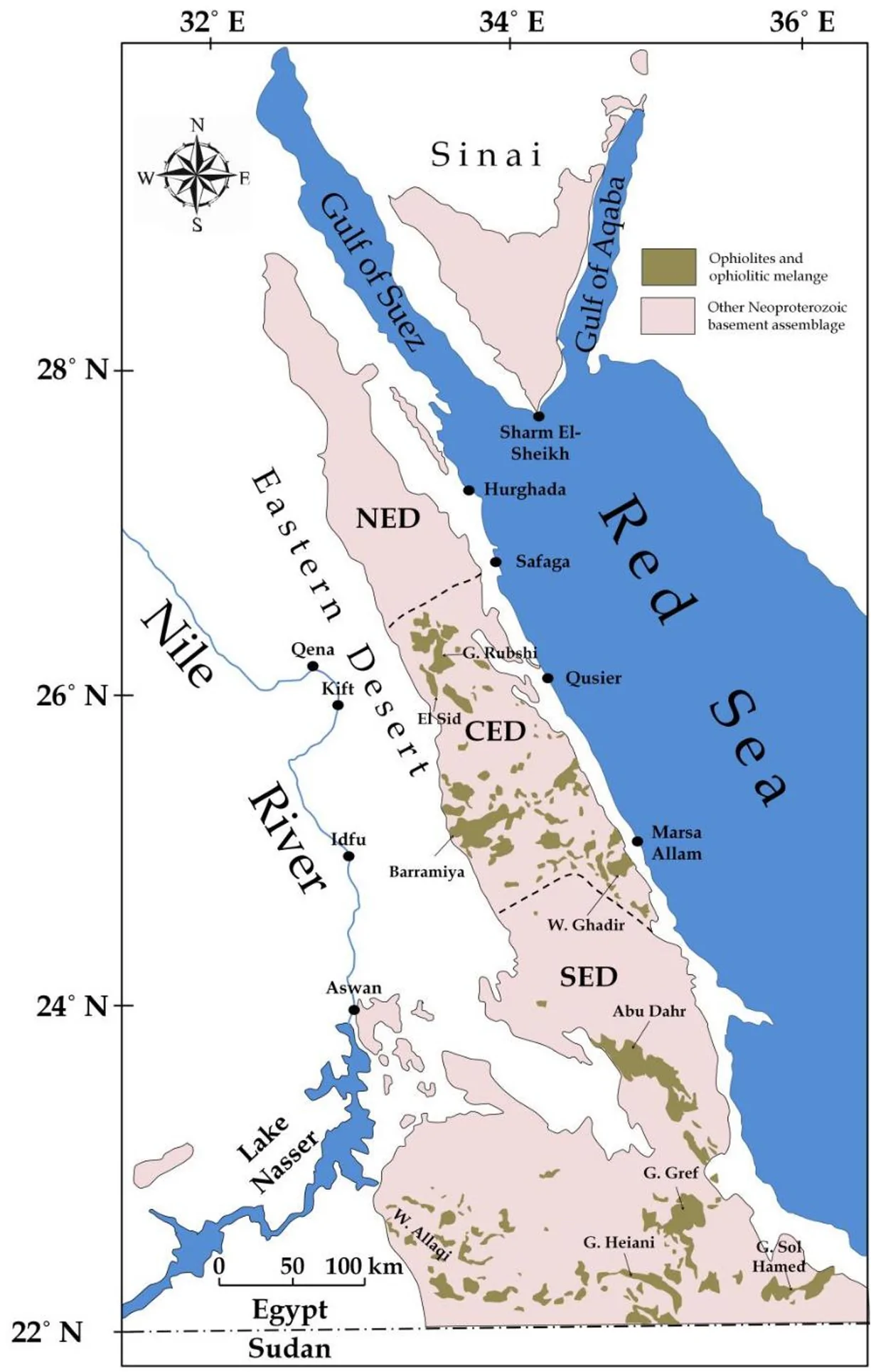
Outcrops of the ophiolites and ophiolitic mélange rocks in the Egyptian Eastern Desert, modified after^44,52,53^. *The map layout and graphical editing were prepared by the authors using CorelDRAW Graphics Suite (Version X7*,* Corel Corporation*,* Ottawa*,* ON*,* Canada;* https://www.coreldraw.com).

In this context, ophiolitic rocks investigated in the current study emerge as particularly promising candidates for radiation shielding applications due to their unique mineralogical compositions and physical properties. Ophiolites represent a sequence in the ancient oceanic crust and upper mantle that is composed of mafic and ultramafic rocks^42^. The ideal section of ophiolite progresses from bottom to top as follows: ultramafic tectonites including peridotite, harzburgite, and dunite, a cumulate gabbro, sheeted dike complex, volcanic rocks (typically basalt with pillow lavas), and finally, sedimentary cover^43^. However, the complete section is rare but exciting as a chaotic mixture and fragments known as ophiolitic mélange that have been tectonically emplaced or obducted on the continental margins, typically found in orogenic belts and suture zones. Ophiolitic rocks are widely distributed in the Eastern Desert of Egypt within the Neoproterozoic rocks, particularly in the central and southern parts, as dismembered fragments and mélanges within volcano-sedimentary assemblages, indicating significant tectonic deformation and metamorphism. Ophiolites in Egypt typically include rock units like serpentinites, metagabbros, metavolcanics, and sheeted dike complexes. The most popular occurrences of ophiolites in the Eastern Desert include Mersa Alam-Idfu Road, Fawakhir, Al-Barramiya, Wadi Ghadir, Gabal Abu Dahr, and Kab Amiri (Fig. 1). They include various types of ultramafic rocks (serpentinites derived from peridotites, pyroxenites, and dunites), mafic rocks (gabbros and diabases), sheeted dykes, metabasalts, and pillow lavas^44–51^.

A considerable body of geological research has been conducted on the ophiolites in Egypt, yet limited studies have focused on their industrial applications. Recent studies have demonstrated the efficacy of serpentinites as effective radiation shielding materials^40,54,55^. In the present study, we investigate, for the first time, the potential of various ophiolitic rocks as low-cost materials for radiation shielding. These rocks exhibit significantly higher densities compared to other geological materials, such as granitoids and sedimentary rocks. For instance, the bulk density of serpentinites—the predominant ophiolitic rock—ranges from 2.65 to 2.94 g/cm^3^^56^, indicating their promising suitability for radiation attenuation. The elevated density of these ophiolitic rocks is attributed to their enrichment in iron, magnesium, and chromium, which is reflected in their mineralogical composition, notably the prevalence of serpentine, olivine, pyroxene, talc, and chromite minerals.

### Research significance

The present investigation represents the first comprehensive multi-analytical evaluation of ophiolitic rocks from the Marsa Alam–Idfu sector of the South Eastern Desert of Egypt for potential radiation shielding applications. Unlike previous studies that primarily focused on isolated rock types or solely on gamma-ray attenuation behavior, this work systematically examines multiple lithological members of the Neoproterozoic ophiolitic sequence, including mafic metavolcanics (MMV), ophiolitic metasediment schist (MMS), serpentinized peridotites (SP), and hornblende metagabbro-diorite (HGD). The study integrates detailed petrographic, mineralogical, geochemical, and thermal characterization using optical microscopy, X-ray diffraction (XRD), thermogravimetric analysis (TGA), and Fourier-transform infrared spectroscopy (FTIR), coupled with experimental gamma-ray attenuation measurements, Monte Carlo (MC) simulations, and neutron shielding assessment.

The novelty of this study lies in establishing direct relationships between mineralogical assemblages, bulk geochemistry, thermal stability, and both gamma-ray and fast-neutron attenuation performance. Particular emphasis is placed on evaluating naturally occurring Egyptian ophiolitic rocks rich in Fe–Mg-bearing minerals, which have received little attention in the radiation shielding literature despite their abundance throughout the Arabian-Nubian Shield. By combining experimental validation with advanced computational modeling, this study provides a robust framework for identifying the key compositional and mineralogical factors governing shielding efficiency and introduces ophiolitic rocks as sustainable, low-cost, and environmentally friendly alternatives to conventional shielding materials.

## Materials

### Geological setting

**Fig. 2:**
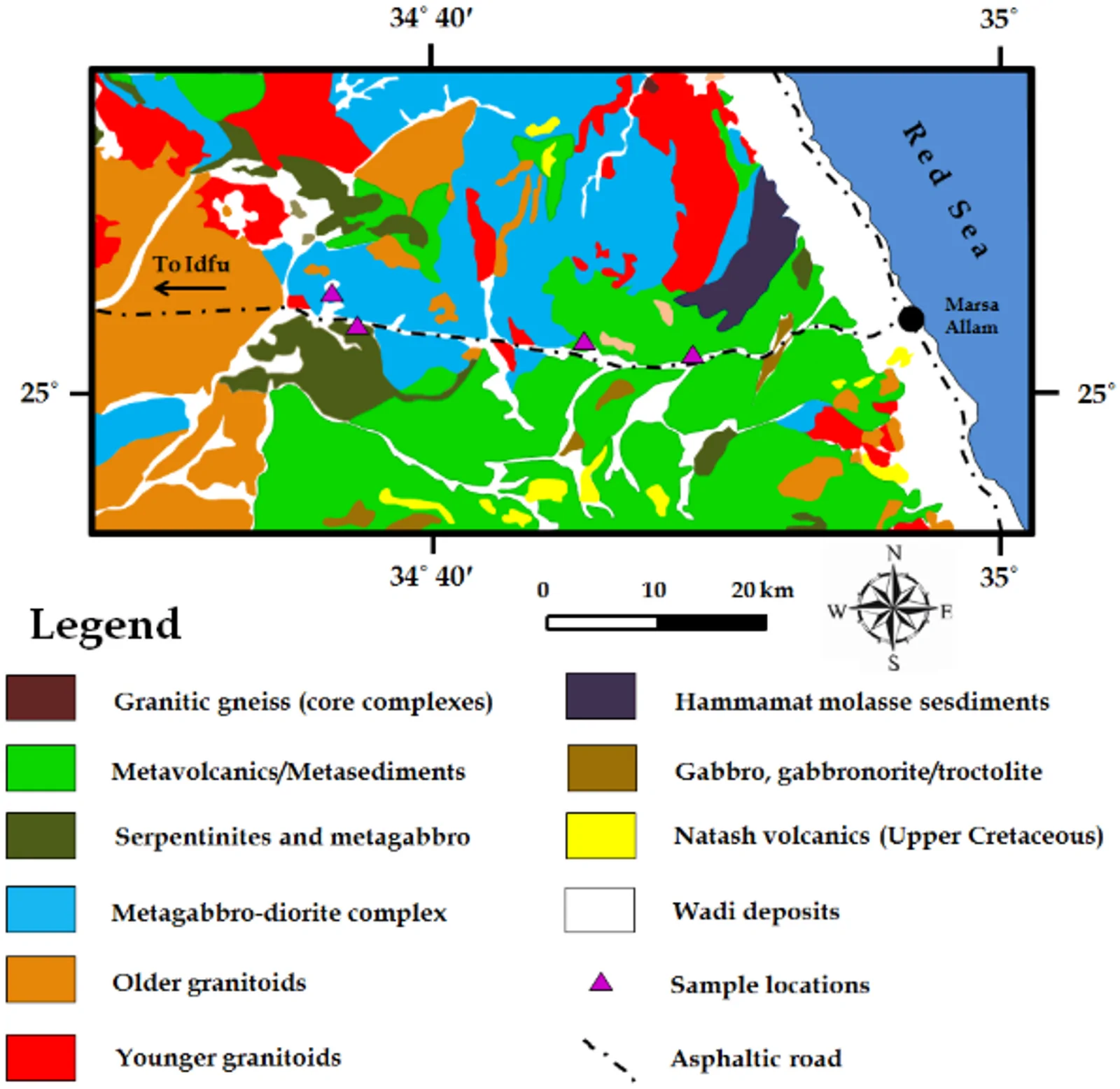
Geological map of the area around Marsa Allam – Idfu road, modified after^57,58^. *This map was prepared by the authors using CorelDRAW X7.*

The rocks selected for the investigation of their radiation shielding properties were sourced from the exposed crystalline basement rock units along the Mersa Alam-Idfu Road in the southern Eastern Desert of Egypt (Fig. 2). These hard rocks represent the northwestern segment of the Arabian-Nubian Shield (ANS), which is geochronologically dated to the late Precambrian (Neoproterozoic) era. The rock units in this area can be arranged in a geochronological sequence from oldest to youngest as follows: granitic gneisses, metasediments, metavolcanics, metagabbro-diorite complex, older granitoids, serpentinites, younger granitoids, Hammamat group sediments, younger gabbro, Natash volcanics, and Wadi deposits.

**Fig. 3:**
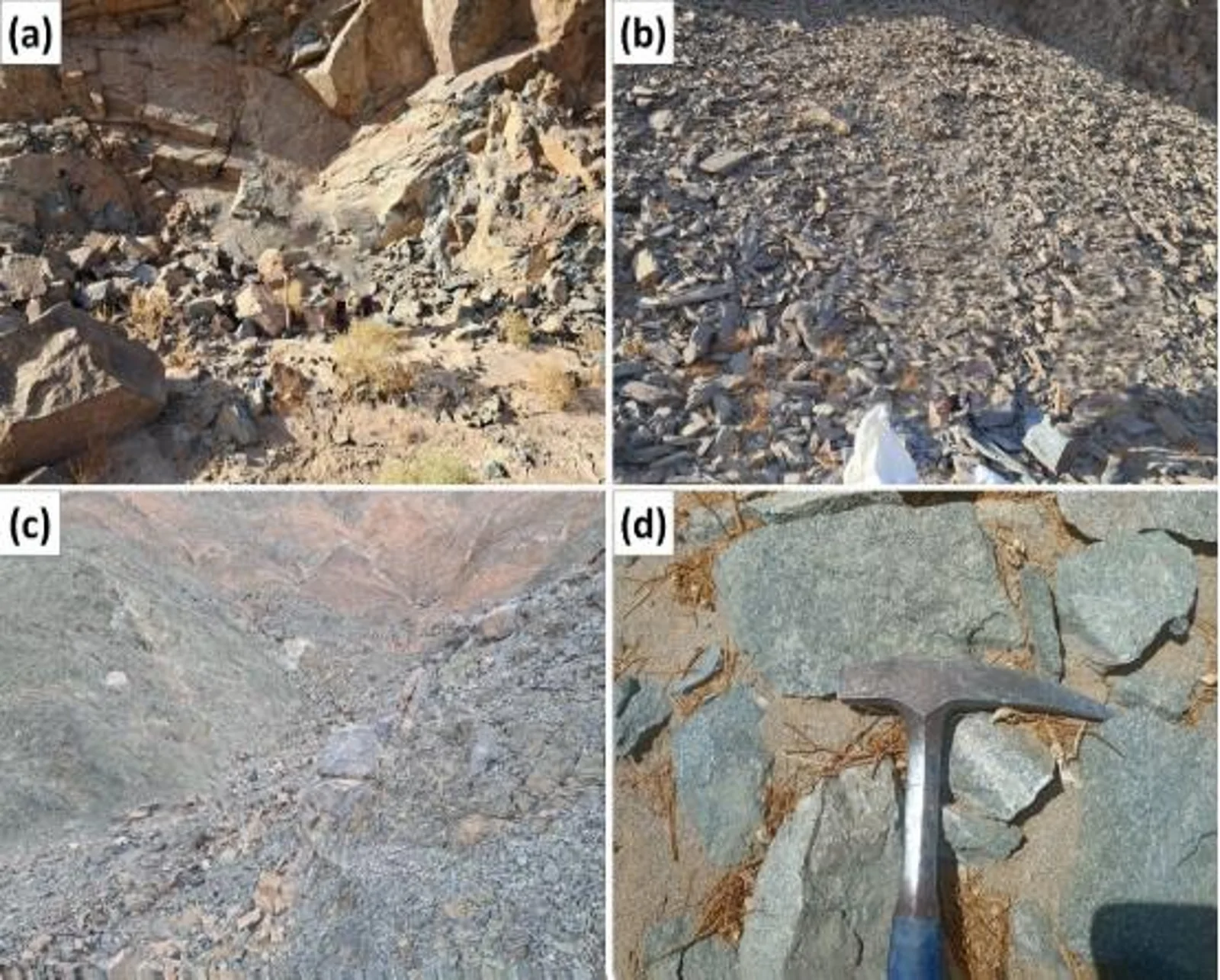
Field photographs for the ophiolitic rocks used in this study, (a) Mafic metavolcanics; (b) ophiolitic metasediment (schist) showing prominent foliation; (c) serpentinized peridotites with interbedded listwanite carbonate bands; (d) hornblende gabbro of the metagabbro- diorite complex.

Interbedded metasedimentary and metavolcanic rocks are widely exposed along the Marsa Alam–Idfu road as isolated peaks and discontinuous ridges (Fig. 3a). They occur mainly as volcanic flows, sills, and thick sheet-like bodies associated with ophiolitic mélanges and island-arc assemblages, and were metamorphosed under greenschist- to amphibolite-facies conditions. The metasediments, derived mainly from volcanogenic mudstones and claystones, include chlorite, epidote, biotite, graphite, hornblende, and tremolite–actinolite schists, together with metagreywackes and slates (Fig. 3b). Metavolcanics range from metabasalts to metarhyolites, with metaandesite being dominant. These rocks are interpreted to have formed in arc and back-arc basin settings, locally associated with ironstone bands^59,60^.

Serpentinites and related rocks form a major part of the ophiolitic sequence along the Marsa Alam–Idfu road. They occur as ENE-trending elongated bodies associated with suture zones and thrust faults (Fig. 3c). The assemblage includes serpentinite, metagabbro, talc–tremolite–carbonate rocks, and talc–magnesite rocks. Mineralogically, antigorite dominates, with minor chrysotile and lizardite. These rocks resulted from serpentinization of ultramafic and mafic protoliths, mainly harzburgite, dunite, and gabbro, likely derived from a forearc mantle wedge setting^47^.

Metagabbro–diorite complexes are widely distributed north of the Marsa Alam–Idfu road and commonly occur in close association with serpentinites as dismembered ophiolitic fragments. The Wadi Dabr suite includes pyroxene–hornblende gabbro, hornblende gabbro (Fig. 3d), quartz–hornblende gabbro, metagabbro, and amphibolite^61^. These rocks are massive, lack primary layering, and show strong tectonic deformation marked by folding and stretching lineations. They were affected by hybridization, deuteric alteration, regional metamorphism, and subsequent intrusion of synorogenic granitoids^62^.

Other rock units in the area include arc-related I-type older granitoids^63,64^ and post-collisional A-type younger granitoids^65,66^. Minor exposures of Hammamat molasse sediments, younger gabbroic intrusions, and Upper Cretaceous Natash volcanic rocks also occur. In addition, Quaternary Wadi deposits composed mainly of sand, pebbles, and boulders are distributed along the main drainage channels and tributaries.

### Petrography

#### Mafic metavolcanics (Metabasaltic andesite)

**Fig. 4:**
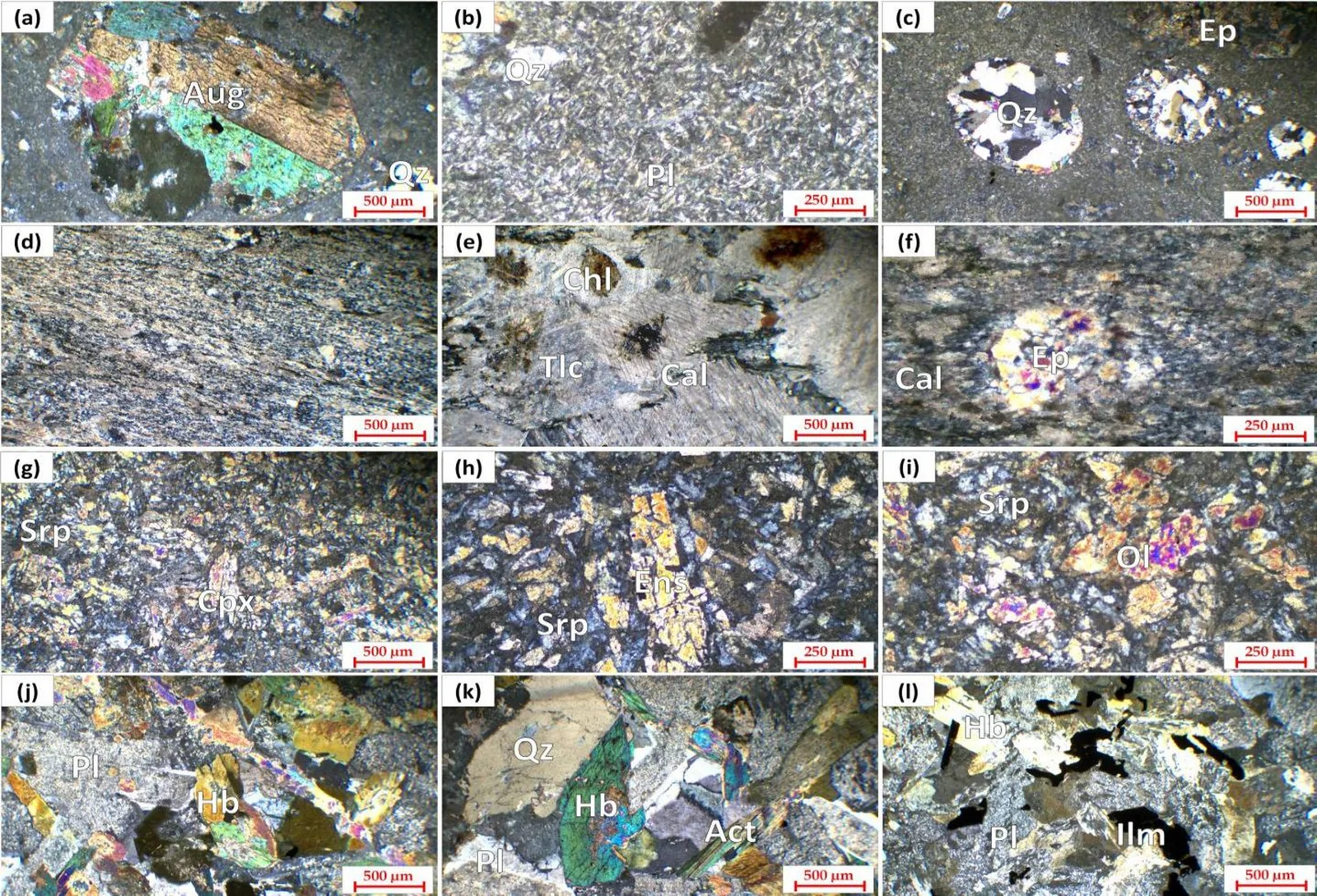
Microphotographs for the ophiolitic rocks (a-c, mafic metavolcanics; d-f, ophiolitic metasediment (schist); g-i, serpentinized peridotites; j-l, hornblende metagabbro-diorite) showing: a) large augite (aug) porphyroblast in a fine groundmass; (b) the fine groundmass composed of fine plagioclase (Pl) laths, pyroxene, quartz (Qz), and epidote; (c) rounded vesicles filled with secondary quartz and agglomerates of epidote (Ep); (d) well-developed foliation by quartz, calcite, muscovite, and talc in metasediments; (e) calcite (Cal), talc (Tlc), and chlorite (Chl) forming thick folia; (f) agglomerates of epidote in a matrix of calcite; (g) serpentine (Srp) and clinopyroxene (Cpx), the main component of serpentinized peridotites; (h) prismatic enstatite (Ens) crystal in a matrix of serpentine; (i) olivine porphyroblasts in a matrix of serpentine; (j) sericitized plagioclase and hornblende (Hb) the main constituents of hornblende gabbro-diorite; (k) secondary actinolite (Act) replacing hornblende; (l) high abundance of the opaque mineral ilmenite (Ilm) in metagabbro.

The mafic metavolcanic rocks are primarily composed of plagioclase, amphibole, and augite, with secondary mineral assemblages including quartz, epidote, actinolite, calcite, and chlorite (Figs. 4a-c). Plagioclase commonly occurs as divergent laths and exhibits partial to complete alteration to sericite and/or epidote. Augite is found as agglomerates or large twinned porphyroblasts embedded in fine ground mass (Fig. 4a), partially transformed into chlorite, epidote, and actinolite. A secondary quartz fills rounded vesicles formed by later silica rich solutions (Fig. 4c). The groundmass comprises micro- to cryptocrystalline plagioclase laths (Fig. 4b), accompanied by lesser amounts of microcrystalline augite, epidote, chlorite, and secondary amphibole. Opaque minerals, especially magnetite, are abundant constitute more than 2% of the modal composition of the metabasaltic andesite. The rocks display various textures, including intergranular, vesicular, ophitic, and porphyritic types. Vesicles are typically infilled with deuteric minerals such as quartz, calcite, chlorite, and epidote.

#### Ophiolitic metasediment (schist)

The Ophiolitic schist within the ophiolitic mélange along the Marsa Allam – Idfu road exhibits a well-developed schistosity, characterized by the alignment of calcite, chlorite, muscovite, talc, and quartz (Fig. 4d-f). The majority of the grains are fine in size, with the exception of some larger calcite crystals (Fig. 4e). These features likely result from the metamorphism of basaltic parent rocks under conditions corresponding to the low-grade greenschist facies. Calcite and talc constitute the dominant rock-forming minerals, comprising approximately 50 modal percent of the rock’s volume (Fig. 4e). Epidote, chlorite, and sericite are identified as the primary secondary minerals present (Fig. 4e, f). The ferromagnesian minerals like chlorite and talc contribute for enhancing radiation shielding characteristics.

#### Serpentinized peridotites

The serpentinized peridotites are primarily composed of relics of orthopyroxene (predominantly enstatite), clinopyroxene (mainly augite), and olivine, variably replaced by serpentine group minerals (Fig. 4g-i). Accessory phases include variable amounts of talc, carbonates, epidote, magnetite, chromite, and tremolite. Serpentine minerals, which commonly replace olivine and pyroxenes—particularly along grain boundaries—exhibit a characteristic mesh texture (Fig. 4h, i). Finely disseminated magnetite, interpreted as of primary origin, is widespread throughout the rock matrix. Talc typically occurs as minute, colorless aggregates replacing antigorite, and may appear as micro-veinlets. Large veins of carbonate minerals, including magnesite and calcite, are distributed throughout the serpentinite. Chromite is present interstitially as rounded, fine-grained crystals exhibiting deep red to reddish-brown coloration.

#### Hornblende metagabbro-diorite

The hornblende gabbro is an equigranular, coarse-grained rock primarily composed of plagioclase and hornblende, with minor biotite and quartz content (Fig. 4j-l). Plagioclase has undergone significant alteration to sericite and epidote (Fig. 4j), while hornblende displays well-developed cleavage, intersecting at approximately 124°. Hornblende constitutes more than 40% of the rock’s modal composition. Accessory minerals include apatite, sphene, and ilmenite, with ilmenite occurring abundantly as skeletal grains (Fig. 4l). This high abundance of hornblende and ilmenite significantly contributes to the γ-radiation attenuation properties of the rock. Secondary calcite formation is commonly observed, resulting from the alteration of calcic plagioclase.

### Geochemistry

#### Mafic metavolcanics

**Fig. 5:**
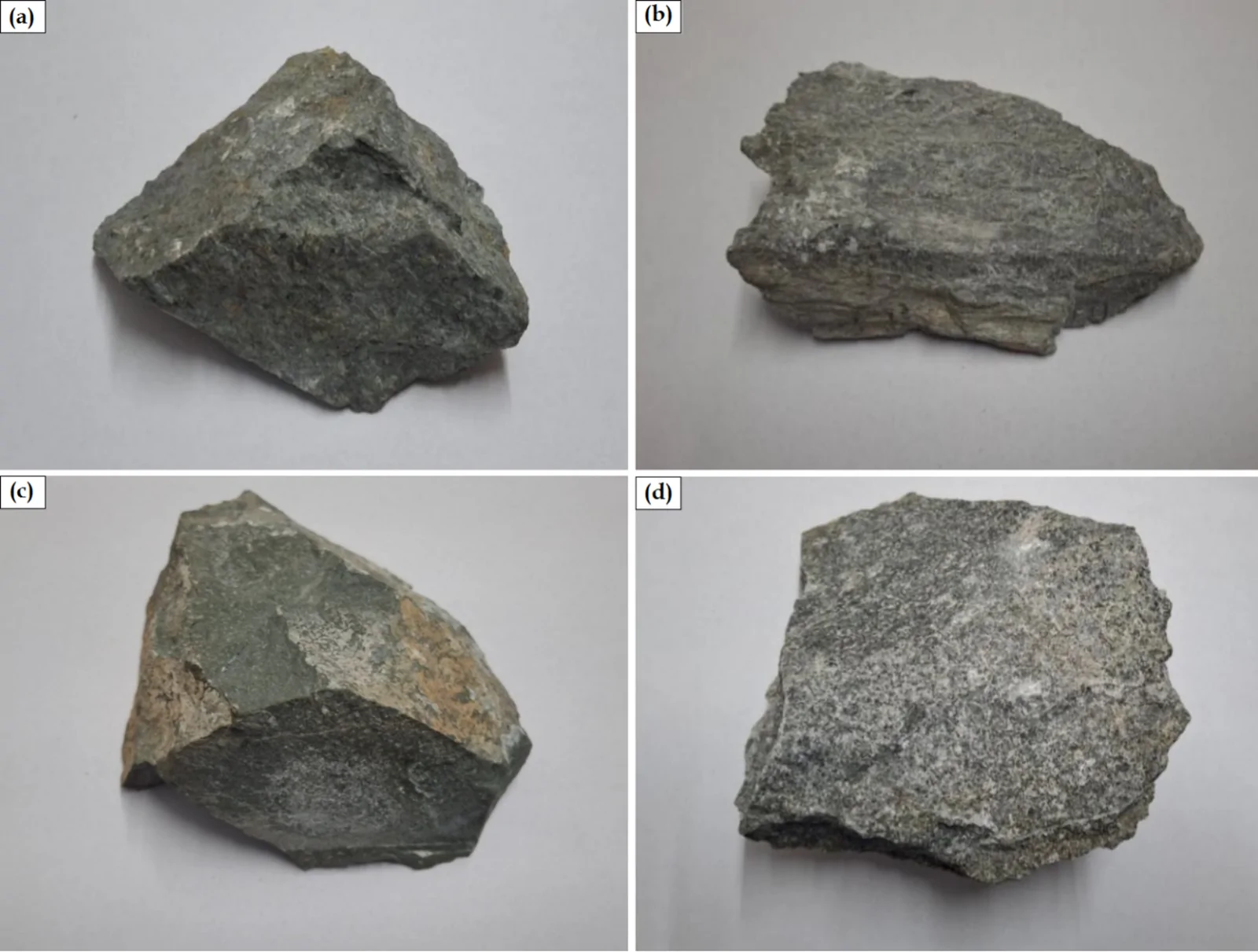
Illustrative images of the rock samples under investigation: (a) Mafic metavolcanics, (b) Ophiolitic metasediment (schist), (c) Serpentinized peridotites, and (d) Hornblende gabbro-diorite.

The analyzed mafic metavolcanic rock (Fig. 5a) is characterized by a low SiO_2_ content (52.82 wt%), consistent with its mafic composition. It can be classified as basaltic andesite according to TAS classification diagram of^67^. The rock is peraluminous, with a high Al_2_O_3_ concentration (15.25 wt%), exceeding the combined content of Na_2_O, K_2_O, and CaO (i.e., Al_2_O_3_ > Na_2_O + K_2_O + CaO), which collectively constitute 10.56 wt%. Elevated FeO (10.73 wt%) and MgO (7.76 wt%) contents indicate a significant presence of ferromagnesian minerals which supports the enhanced attenuation behavior observed for this sample. Other oxides, including P_2_O_5_, MnO, and TiO_2_, are present in low concentrations, ranging from < 0.2 to 0.8 wt% (Table 1).

#### Ophiolitic metasediment (schist)

The ophiolitic schist, as shown in (Fig. 5b), exhibits a geochemical composition closely comparable to that of the metavolcanic rocks, indicating a shared origin or derivation from metavolcanic precursors. The rock is characterized by a low SiO_2_ content (51 wt%), consistent with its mafic nature. Al_2_O_3_ content is notably high (24.32 wt%), primarily reflecting the abundance of phyllosilicate minerals, such as mica and chlorite. The moderate concentrations of FeO (6.15 wt%) and MgO (1.99 wt%) are attributed to the presence of iron- and magnesium-bearing minerals such as chlorite, biotite, and talc, although these values are the lowest among the studied ophiolitic rocks, likely due to the limited presence of ferromagnesian phases and the absence of serpentine or magnesium carbonates. CaO is influenced by the presence of calcite, while the relatively high combined alkali content (Na_2_O + K_2_O > 6.8 wt%) reflects contributions from alkali-bearing clay minerals. Minor oxides, including P_2_O_5_, TiO_2_, and MnO, are present in low concentrations (Table 1).

#### Serpentinized peridotites

The analyzed serpentinized rock (Fig. 5c) shows the highest value of loss on ignition (LOI), which is attributed to elevated crystal water retained in hydrous minerals, primarily serpentine. The rock exhibits high MgO (18.47 wt%) and CaO (9.45 wt%) contents, alongside a notably low SiO_2_ content (31.08 wt%), consistent with its ultramafic composition. The elevated CaO concentration is associated with the formation of secondary carbonates (listwanite) during serpentinization processes. Magnesium content exceeds that of iron, as reflected by a high Mg# value [Mg/(Mg + Fe)] of approximately 0.7. Al_2_O_3_ is also abundant (17.55 wt%), surpassing the combined content of alkalis and CaO (totaling 10.56 wt%). Alkali oxides (Na_2_O and K_2_O) are present in very low concentrations (less than 0.2 wt% combined), which is typical for serpentine-rich ultramafic rocks. TiO_2_ content is relatively high, suggesting the presence of Ti-bearing minerals such as rutile and ilmenite which supports the enhanced attenuation behavior observed for this sample.

The SO_3_ concentration is elevated (3.22 wt%), indicating the likely presence of sulfide minerals, including pyrite (FeS_2_) and chalcopyrite (CuFeS_2_). Other oxides, including P_2_O_5_ and MnO, are present in minor amounts (~ 0.2 wt%) (Table 1).

#### Hornblende gabbro-diorite

The hornblende gabbro sample (Fig. 5d) collected from Wadi Dabr is characterized by a relatively low silica content (55.96 wt%), and is classified as gabbroic diorite based on the TAS classification diagram^68^. This rock exhibits elevated concentrations of Al₂O₃ (16.84 wt%), FeO (8.91 wt%), CaO (7.31 wt%), Na_2_O (3.75 wt%), and MgO (3.26 wt%). The high FeO and MgO contents reflect the abundance of ferromagnesian minerals, whereas the elevated CaO and Na_2_O are primarily attributed to the presence of hornblende [Ca, Na)_2_-₃(Mg, Fe, Al)₅(Al, Si)_8_O_22_(OH, F)_2_] and calcic plagioclase [(Ca, Na)(Al, Si)AlSi_2_O_8_]. The TiO_2_ content is relatively high, suggesting the presence of titanium-bearing accessory minerals such as rutile and ilmenite. P_2_O_5_ concentration is the highest among the analyzed ophiolitic rocks, consistent with the presence of apatite. MnO occurs in minor amounts (~ 0.16 wt%) (Table 1).

**Table 1:** Major oxide composition of the studied ophiolitic rocks.

	Mafic metavolcanic	Ophiolitic metasediment	Serpentinized peridotites	Hornblende gabbro-diorite
SiO_2_	52.82	51	31.08	55.96
Al_2_O_3_	15.25	24.32	17.55	16.84
CaO	5.99	6.60	9.46	7.32
FeO	10.37	6.15	13.24	8.92
K_2_O	0.76	4.77	0.133	1.04
Na_2_O	3.82	2.13	0.02	3.75
MgO	7.76	1.99	18.47	3.27
MnO	0.22	0.13	0.297	0.168
P_2_O_5_	0.298	0.16	0.252	0.367
TiO_2_	0.784	0.617	1.90	0.80
LOI	1.93	2.12	6.6	1.50
Total	100	99.98	98.99	99.94
Density (g/cm^3^)	2.79	2.74	3.00	2.73

Note: Total oxides below 100 wt%, particularly in serpentinized samples, are primarily attributed to unanalysed trace elements such as Cr, Ni, and Co.

## Methods

### XRD

X-ray diffraction (XRD) analysis was conducted on four representative rock types to identify their mineralogical compositions and crystalline phases. The obtained diffraction patterns reveal distinctive mineral assemblages that are consistent with the chemical compositions and petrographic observations of the investigated samples. The XRD results demonstrate clear mineralogical signatures for each rock type that reflect their different petrogenetic origins and metamorphic histories. The mafic metavolcanics exhibit typical greenschist-facies mineral assemblages, whereas the ophiolitic schist indicates polyphase metamorphism of mixed protoliths. In addition, the serpentinized peridotites display extensive hydrothermal alteration of ultramafic rocks, while the hornblende gabbro-diorite represents relatively fresh igneous mineralogy with minor secondary alteration phases. The XRD measurements were performed using a PANalytical X-ray diffractometer (Panalytical B.V., Netherlands; ISO 9001/14001 KEMA certified) equipped with a Cu anode X-ray tube (Cu-Kα radiation). The instrument was operated at a tube voltage of 40 kV and a current of 30 mA. Finely ground powder samples were analyzed using X’Pert High Score Software 5.0 for data acquisition and phase identification, while the raw diffraction data were recorded in (*.XRDML) format. Prior to analysis, the instrument alignment and calibration were verified according to the manufacturer’s standard procedures to ensure the reliability and reproducibility of the diffraction measurements.

### TGA

TGA is a widely used technique in geoscience that measures the change in mass of a rock sample as it is heated under controlled conditions^69^. This technique is particularly effective for evaluating the thermal stability and decomposition behavior of minerals containing volatile constituents, such as structural water, hydroxyl groups, carbonates, and sulfides. In the case of ophiolitic rocks, TGA provides valuable information regarding the presence and thermal decomposition of hydrous minerals, carbonate phases, and alteration products, which significantly influence the thermal stability and durability of the rocks under elevated temperature conditions. The thermal analysis was carried out using a LINSEIS STA PT1600 simultaneous thermal analyzer. Prior to testing, the rock samples were finely powdered to ensure thermal homogeneity. Approximately suitable amounts of each sample were placed in aluminum crucibles and heated from room temperature up to 1000 °C at a constant heating rate of 10 °C/min under controlled atmospheric conditions. During the analysis, the instrument continuously recorded temperature, mass loss, and derivative weight change (DTG), enabling detailed evaluation of the thermal behavior and decomposition stages of the investigated samples. The instrument was calibrated according to the manufacturer’s standard calibration procedures before measurements to ensure the accuracy and reproducibility of the obtained results.

### FTIR analysis

Fourier Transform Infrared (FTIR) spectroscopy provides direct evidence of the functional groups and lattice vibrations that reflect the mineralogical composition of the studied rocks. A comparative examination of the FTIR spectra reveals clear differences that are directly linked to the dominant mineralogical and chemical features of each rock type^70^. The identified absorption bands provide valuable information regarding the presence of silicates, hydroxyl-bearing minerals, carbonates, and other secondary alteration products within the different ophiolitic rock types. The FTIR spectra were obtained using a BRUKER Tensor 37 FTIR spectrometer over the wavenumber range of 400–4000 cm^-1^. Finely powdered samples were prepared prior to analysis to ensure spectral homogeneity and reliable signal acquisition. The obtained spectra were used to identify the characteristic vibrational bands corresponding to the major mineral phases present in the investigated samples. Instrument calibration and spectral acquisition procedures were conducted according to the manufacturer’s recommended operating conditions to ensure the accuracy and reproducibility of the measurements.

### Radiation shielding analysis

**Fig. 6:**
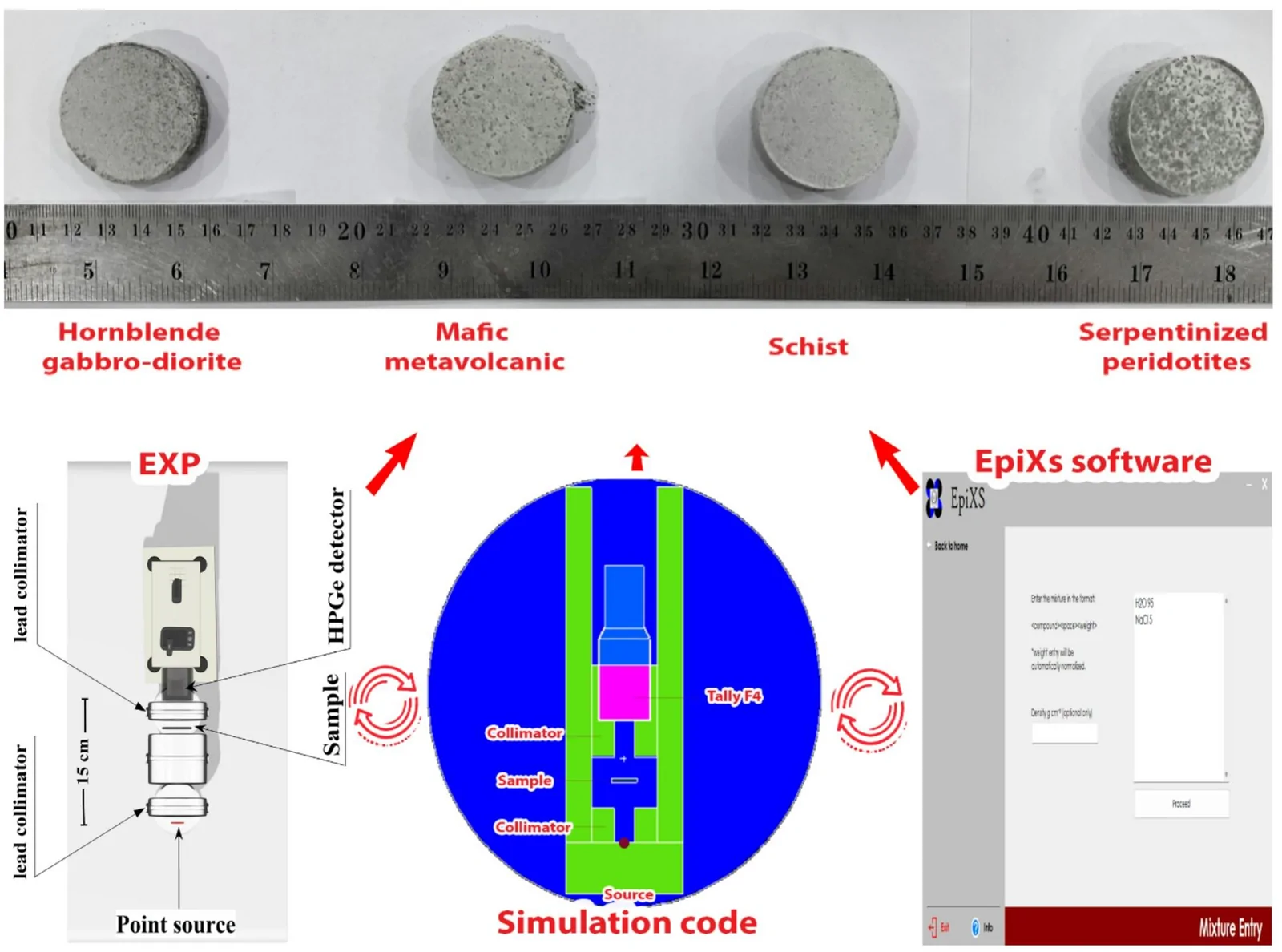
The γ-rays shielding tests for the collected ophiolitic rocks.

To conduct analytical measurements related to the theoretical and practical radiation shielding performance of the samples, they were finely ground and then pressed into discs with a thickness ranging from 1.512 to 1.612 cm and a diameter of 3.9 to 4.21 cm (Fig. 6). The radiation shielding measurements performed were as follows:

#### Practical γ-shielding measurements

The experimental evaluation of the gamma-ray shielding performance of the investigated ophiolitic rocks was carried out using a handheld radioisotope identification device (HRID) incorporating a coaxial p-type high-purity germanium (HPGe) detector. The detector was employed to measure the γ-ray photopeaks of Cs-137 (0.662 MeV) and Co-60 (1.173 and 1.332 MeV). Owing to its superior energy resolution, the HPGe detector represents an effective tool for the precise spectrometric identification and analysis of discrete gamma-ray photopeaks^71^. The acquired spectra were processed using Gamma Vision 6.08 software (https://www.ortec-online.com/products/software/gammavision). The experimental setup consisted of a lead-shielded collimator arrangement designed to minimize scattered radiation and maintain good geometry conditions during attenuation measurements (Fig. 6). The detector was energy calibrated using standard Cs-137 and Co-60 reference sources prior to measurements. A fixed source-to-detector distance of 15 cm was maintained throughout all measurements to ensure good geometry conditions, reduce scattered radiation effects, and provide consistent counting statistics during the attenuation experiments. The total acquisition time for the attenuation measurements was 2400 min, distributed among the investigated gamma-ray energies and experimental runs. The relatively long counting duration was selected to improve counting statistics, minimize statistical uncertainties, and enhance the reliability of the measured attenuation parameters, particularly for low transmitted photon intensities after passing through dense rock samples. The net count rates corresponding to the characteristic gamma-ray energies of Cs-137 and Co-60 were subsequently determined from the recorded spectra^72^.

#### Analytical and simulation code

The computational modeling in this study was performed using the MCNP- Version 5 Monte Carlo code^73^ to simulate gamma-ray transport through the investigated ophiolitic rock samples. The simulation geometry reproduced the experimental arrangement, including the source–detector distance, sample thickness, bulk density, and elemental composition of the investigated rocks obtained from the geochemical analyses^74^. A point gamma-ray source with photon energies ranging from 0.015 to 15 MeV was employed to evaluate the attenuation behavior of the studied materials over a broad energy spectrum.

The geometric configuration implemented in the MC model is illustrated in Fig. 6, which presents both the simulation setup and the corresponding experimental arrangement^75^. The detector was positioned within a lead collimator to minimize scattered radiation and maintain good geometry conditions during the attenuation measurements. Photon flux calculations were performed using the F4:P tally card for gamma-ray transport analysis^76^. The attenuation parameters were calculated according to the Beer–Lambert law as follows^77–80^:

1 $${\mathrm{I}}\,=\,{{\mathrm{I}}_{\mathrm{o}}}{{\mathrm{e}}^{( - }}^{{\mu .{\mathrm{t}})}}$$

2 $${\mathrm{HVL}}={\text{}}\frac{{{\mathrm{ln}}2}}{\mu }$$

3 $${\mathrm{TVL}}=3.32{\mathrm{HVL}}$$

where I₀ represents the incident photon intensity before interaction with the shielding material, while I corresponds to the transmitted photon intensity after passing through a material thickness (t). The linear attenuation coefficient (µ) describes the probability of photon interaction per unit path length within the absorber material^81^. The half-value layer (HVL) and tenth-value layer (TVL) represent the material thicknesses required to reduce the incident gamma-ray intensity to 50% and 10%, respectively. Lower HVL and TVL values indicate superior shielding performance. To validate the simulation results, the calculated attenuation parameters were cross-verified using the EpiXs 2.0.1 software package^82^. The software, available at (https://www.pnri.dost.gov.ph/index.php/downloads/software/epixs), was employed to determine the mass attenuation coefficient (MAC), effective atomic number (Zeff), and related shielding parameters using the EPICS2017 nuclear database^83^.

To evaluate the fast neutron shielding performance of the selected mafic and ultramafic rock samples, their macroscopic fast neutron removal cross-section (FCS), half-value layer (HVL), and relaxation length (λ) were determined. These parameters constitute the standard framework for evaluating radiation shielding materials. The FCS dictates the probability per unit path length that a fast neutron is removed from the primary beam through colliding interactions (scattering and absorption), thus directly reflecting attenuation capacity. Meanwhile, the HVL specifies the precise thickness required to reduce the initial neutron intensity by half, serving as a pivotal parameter in radiation protection engineering. Lastly, λ values characterizes the distance over which the neutron flux undergoes exponential decay within the medium. In general, enhanced shielding performance is dictated by elevated FCS values coupled with minimized HVL and λ dimensions. The neutron shielding parameters were calculated using the Phy-X/PSD software, a widely validated radiation shielding platform that enables rapid and reliable determination of neutron and photon interaction parameters based on elemental composition and density data^84^. The software, available online at (phy-x.net/PSD), incorporates established nuclear databases and analytical models, allowing accurate estimation of fast neutron removal cross sections and related shielding characteristics for a wide range of natural and engineered materials.

## Results and discussion

### XRD analysis

#### Mafic metavolcanics

**Fig. 7:**
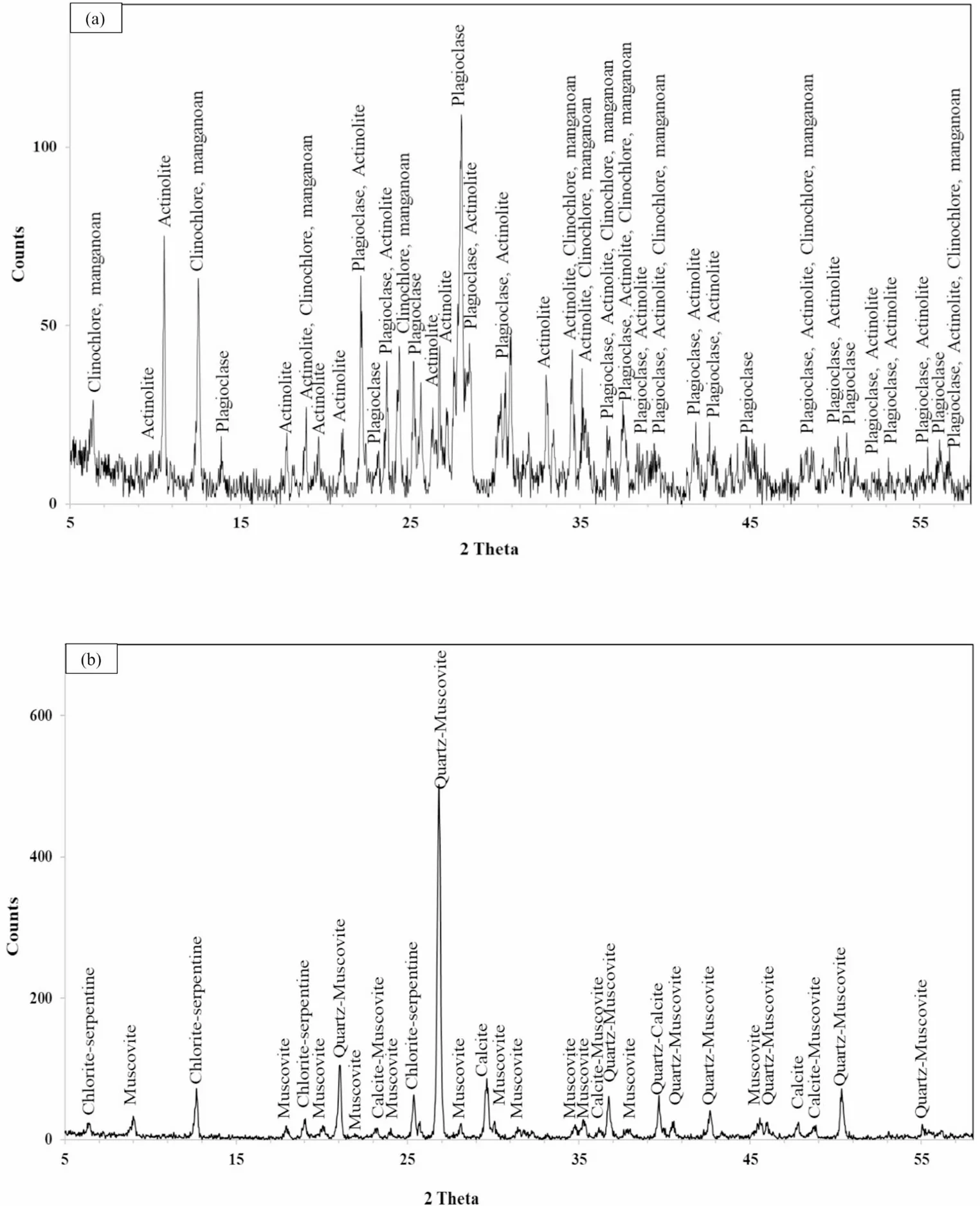

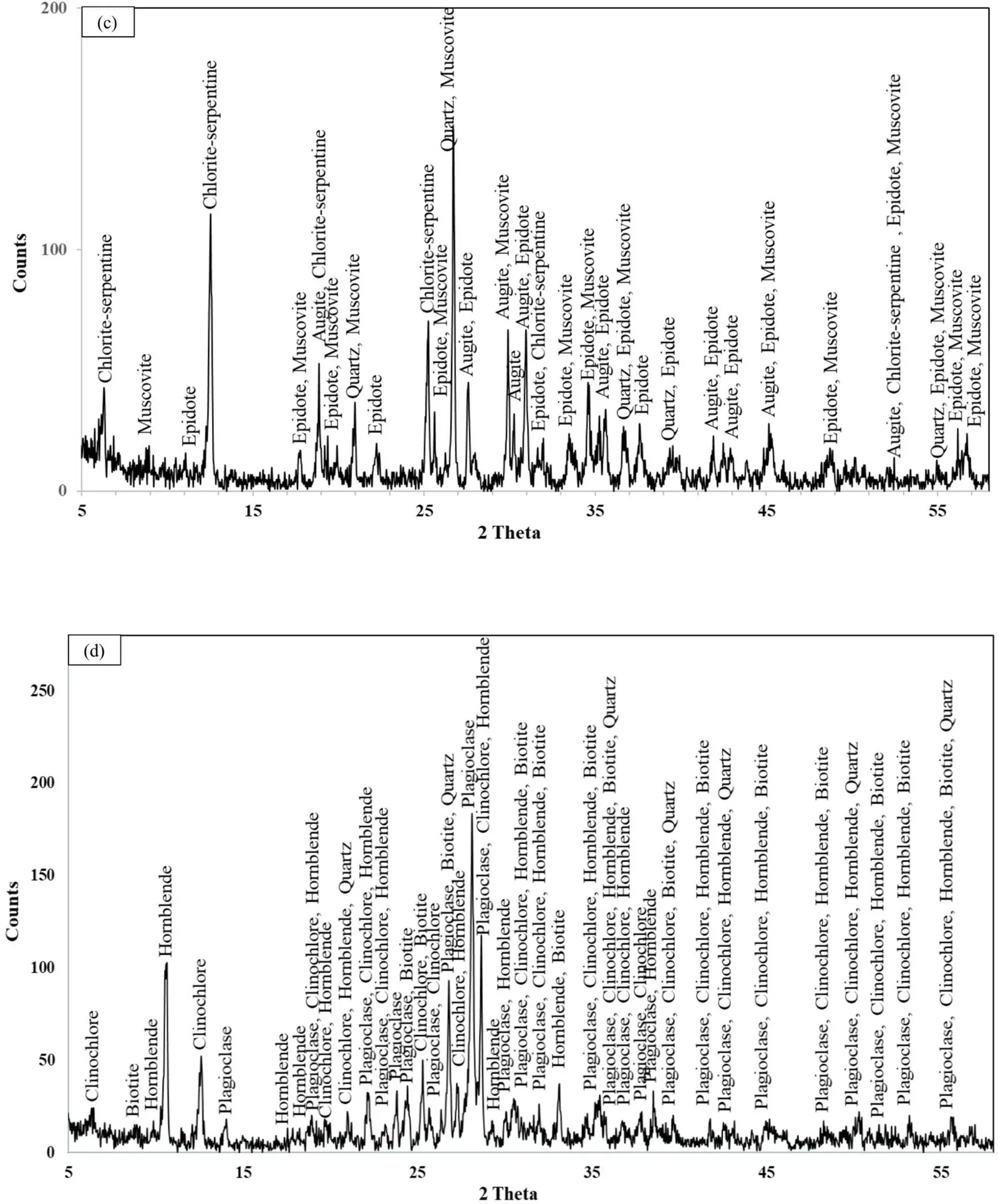
XRD patterns for: (a) Mafic metavolcanics, (b) Ophiolitic metasediment (schist), (C) Serpentinized peridotites, (d) Hornblende gabbro-diorite.

The XRD pattern of mafic metavolcanics (Fig. 7a) reveals a mineral assemblage dominated by plagioclase, actinolite, chlorite (manganoan clinochlore). This mineralogy is consistent with the intermediate SiO_2_ content (52.82 wt%) and moderate Al_2_O_3_ (15.25 wt%) and MgO (7.76 wt%) concentrations. The presence of actinolite reflects the metamorphic transformation of primary pyroxene under greenschist facies conditions, supported by the moderate CaO (5.989 wt%) and FeO (10.37 wt%) contents. Clinochlore formation is attributed to the alteration of mafic minerals, consistent with the elevated MgO and FeO values. The Na_2_O content (3.82 wt%) supports the abundance of plagioclase observed petrographically, while the low K_2_O (0.759 wt%) reflects minimal potassic alteration.

#### Ophiolitic metasediment (Schist)

The XRD analysis of ophiolitic schist (Fig. 7b) identifies quartz, muscovite, chlorite-serpentine, and minor calcite as the primary mineral phases. This assemblage reflects the high Al_2_O_3_ content (24.32 wt%) and elevated K_2_O (4.77 wt%), supporting extensive muscovite development during metamorphism. The moderate SiO_2_ content (51 wt%) is consistent with the presence of quartz, while the relatively low MgO (1.99 wt%) corresponds to limited chlorite formation. The chlorite-serpentine association suggests mixed protoliths of basaltic and ultramafic compositions, typical of ophiolitic mélanges. The low Na_2_O (2.13 wt%) and CaO (6.597 wt%) values reflect the limited preservation of primary igneous minerals, consistent with the advanced alteration and metamorphic recrystallization observed petrographically.

#### Serpentinized peridotites

The serpentinized peridotites exhibit a complex XRD pattern (Fig. 7c) showing quartz, muscovite, chlorite, serpentine, augite, and epidote. This mineral assemblage reflects the distinctive chemical signature with very low SiO_2_ (31.08 wt%) and exceptionally high MgO (18.47 wt%) contents, characteristic of ultramafic rocks. The chlorite-serpentine association dominates the pattern, consistent with extensive serpentinization of primary olivine and pyroxene. Relict augite peaks correspond to preserved clinopyroxene cores observed petrographically. The presence of epidote reflects Ca-Al-rich compositions (CaO: 9.459 wt%, Al_2_O_3_: 17.55 wt%) and is consistent with metamorphic conditions. The high FeO content (13.24 wt%) supports the abundant magnetite formation during serpentinization. The SO_3_ content (3.221 wt%) may reflect sulfide mineralization associated with the ophiolitic environment. The extremely low K_2_O (0.133 wt%) and Na_2_O (0.02 wt%) values are typical of ultramafic protoliths.

#### Hornblende Gabbro-Diorite

The XRD pattern of hornblende gabbro-diorite (Fig. 7d) reveals plagioclase, quartz, hornblende, clinochlore, and biotite as the main mineral phases. This assemblage is consistent with intermediate to felsic composition (SiO_2_: 55.96 wt%) and moderate Al_2_O_3_ content (16.84 wt%). The prominent plagioclase peaks correspond to the high Na_2_O (3.75 wt%) and CaO (7.318 wt%) contents, while biotite formation is supported by the moderate K_2_O (1.036 wt%) content. Hornblende dominance reflects the intermediate MgO (3.267 wt%) and FeO (8.915 wt%) compositions, consistent with calc-alkaline magmatic evolution. The presence of clinochlore indicates low-grade alteration of primary mafic minerals. Quartz occurrence reflects the evolved nature of this rock type within the gabbro-diorite compositional range. The mineral assemblage is consistent with crystallization from hydrous calc-alkaline magmas under moderate pressure conditions.

### TGA analysis

**Fig. 8:**
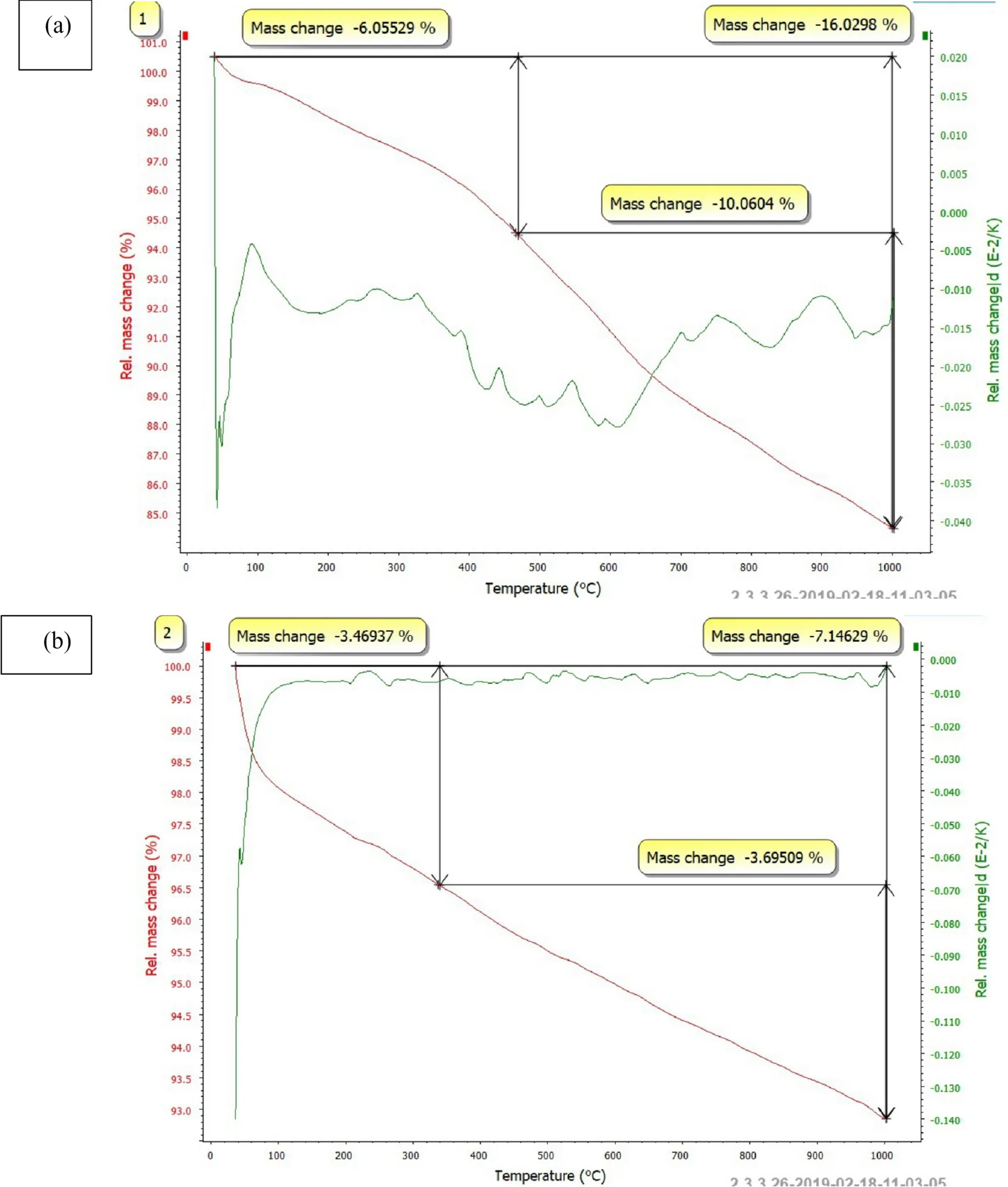

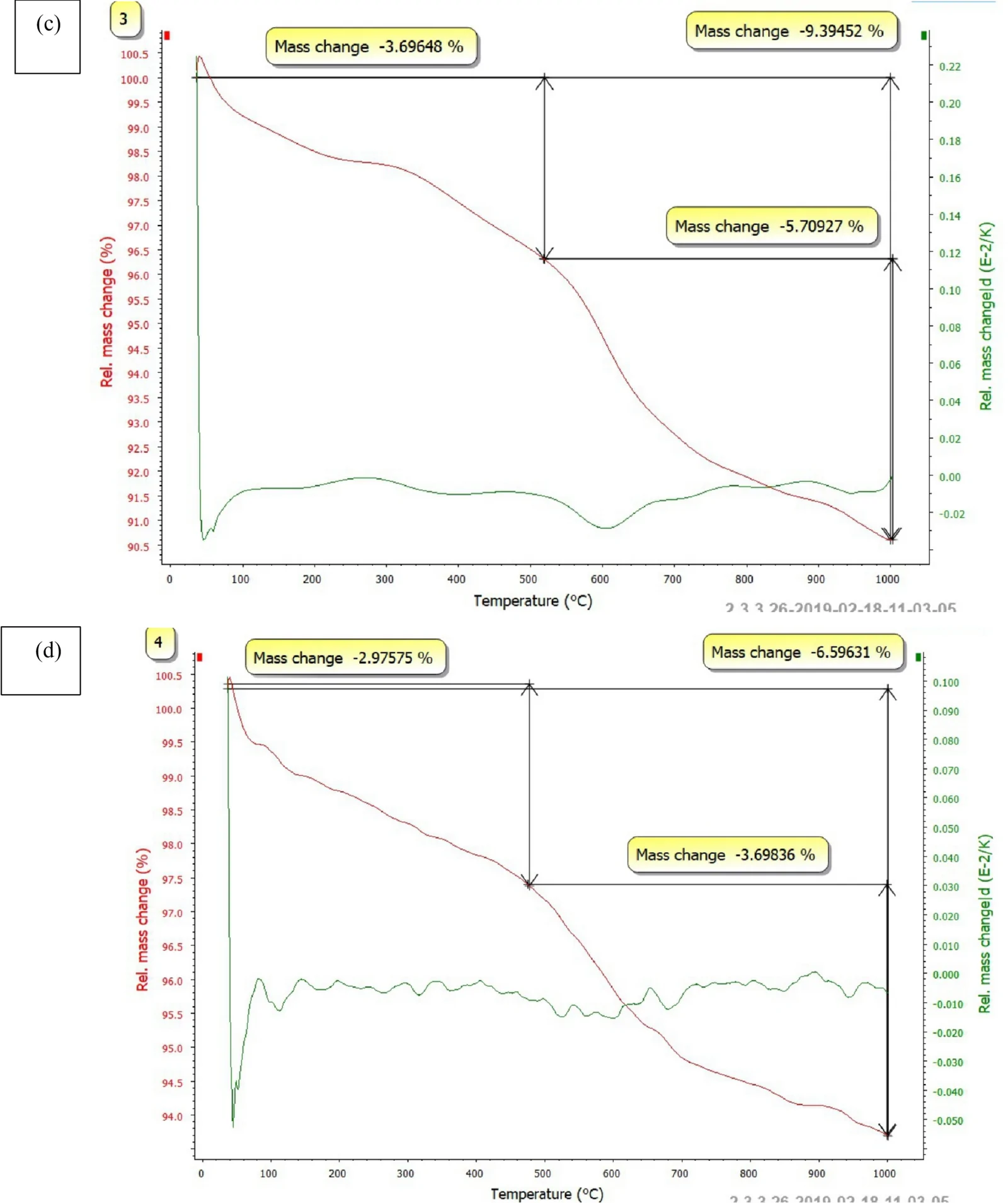
TGA analysis of the rock samples (a) Mafic metavolcanic, (b) Ophiolitic metasediment, (c) Serpentinized peridotites, and (d) Hornblende gabbro-diorite.

The mafic metavolcanic rock (Fig. 8a) recorded the largest weight loss, with about 6% between 50 and 475 °C and about 10% between 475 and 1000 °C, giving a total of approximately 16%. This significant loss can be attributed to the dehydration of alteration minerals like chlorite, epidote and actinolite, as well as the breakdown of secondary calcite, all of which contribute both water and carbon dioxide during heating. The ophiolitic metasediment or schist (Fig. 8b) showed a more moderate total weight loss of around 7%, which occurred in two steps, the first due to dehydration of phyllosilicates at lower temperature and the second due to decarbonation of calcite at higher temperature. The serpentinized peridotite (Fig. 8c) experienced a total weight loss of about 9%, reflecting the combined effect of serpentine dihydroxylation and decomposition of secondary carbonates. This behavior is consistent with its chemical composition rich in magnesium oxide and calcium oxide and its petrography showing abundant serpentine minerals and carbonate veins.

In contrast, the hornblende gabbro-diorite (Fig. 8d) exhibited the smallest total mass loss, about 6.6%, which is consistent with its dominance of coarse-grained plagioclase and hornblende and its relatively low content of hydrous or carbonate phases. The obtained results demonstrate that thermal stability is closely related to mineralogy and bulk chemistry, with rocks rich in hydrous silicates and carbonates showing higher weight losses.

Although serpentine minerals are often regarded as stable at moderately high temperatures before decomposition, their large proportion of bound hydroxyl groups, together with the presence of secondary carbonates, leads to a considerable cumulative mass loss. Therefore, when stability is assessed in terms of total mass retained during heating, the hornblende gabbro-diorite is the most stable, followed by the ophiolitic schist, then the serpentinized peridotite, while the mafic metavolcanic rock is the least stable. It’s worth mentioning that the thermal stability is not a direct measure of the mechanical strength of the rock. Some rocks may be thermally stable but mechanically weak (for example, a coarse crystalline rock with low porosity may show little mass loss but still fracture under stress), while another may be thermally unstable due to volatile release yet mechanically competent at room conditions. In other words, rocks rich in hydrous and carbonate minerals tend to lose mass rapidly when heated, which can weaken their internal cohesion and reduce their strength at high temperatures. In contrast, rocks dominated by anhydrous silicates like plagioclase, pyroxene, and quartz are generally more resistant to thermal degradation, helping to preserve their mechanical integrity at elevated temperatures.

From a radiation shielding perspective, the thermal decomposition processes identified by TGA may have important implications for the long-term durability of shielding materials operating under elevated-temperature conditions. The dehydration of hydrous minerals and the decarbonation of carbonate-bearing phases can generate microstructural defects, increase porosity, and reduce bulk density, all of which may adversely affect both the mechanical integrity and radiation attenuation capacity of the rocks. Since gamma-ray and neutron shielding performance are strongly dependent on density and elemental composition, any thermally induced increase in pore volume or loss of chemically bound constituents may lead to a gradual reduction in shielding efficiency. Consequently, rocks exhibiting lower mass losses, such as the hornblende gabbro-diorite, are expected to maintain their structural stability and shielding effectiveness more successfully during prolonged thermal exposure. In contrast, the higher weight losses observed in the mafic metavolcanic and serpentinized peridotite samples suggest a greater susceptibility to thermally induced alteration under extreme service temperatures. Nevertheless, it should be noted that the major decomposition reactions identified in this study occur predominantly above 400–500 °C, which is significantly higher than the temperatures encountered in most medical, industrial, and conventional nuclear shielding facilities. Therefore, the investigated rocks are expected to remain structurally and radiologically stable under normal operating conditions, while the TGA results provide valuable insight into their behavior under accidental or extreme thermal scenarios.

### FTIR analysis

**Fig. 9:**
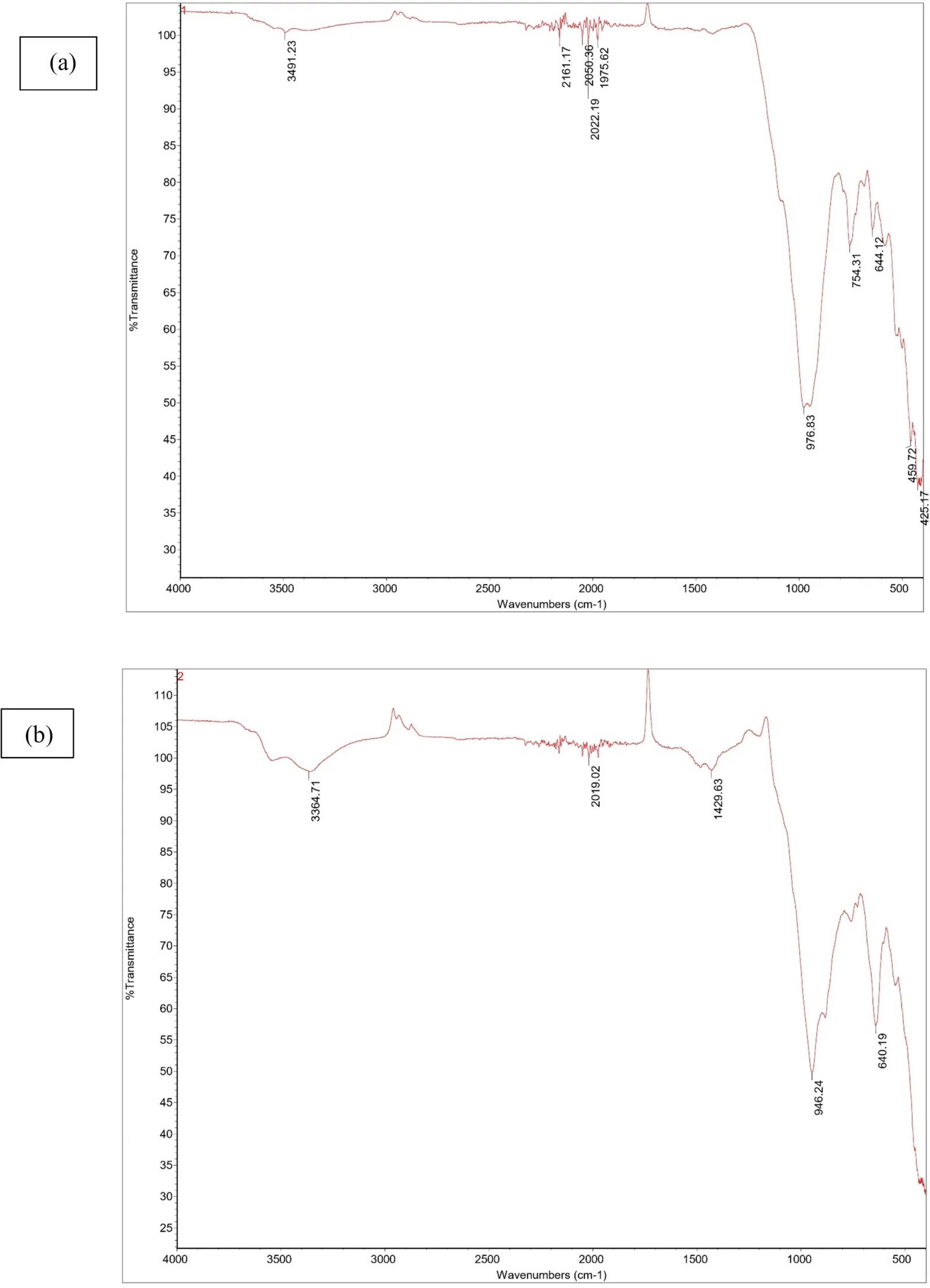

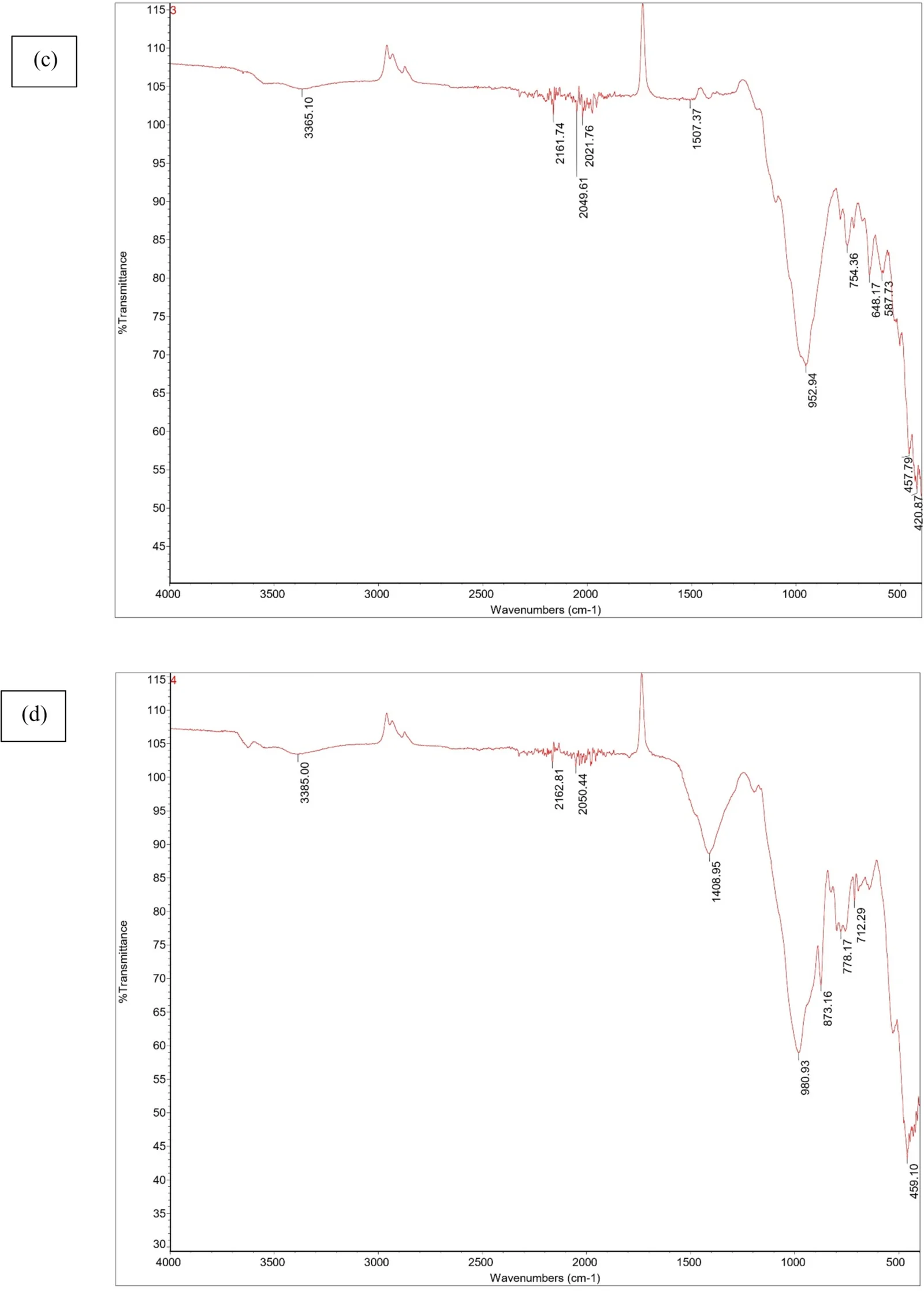
FTIR analysis of the rock samples (a) Mafic metavolcanic, (b) Ophiolitic metasediment, (c) Serpentinized peridotites, and (d) Hornblende gabbro-diorite.

The spectrum of the mafic metavolcanic rock (Fig. 9a) is dominated by strong Si–O stretching bands in the fingerprint region, including peaks near 976, 754, and 644 cm^− 1^, which correspond to the vibrations of plagioclase and augite, the major constituents identified petrographically. A broad OH absorption around 3491 cm^− 1^ indicates the presence of secondary hydrous phases such as chlorite and epidote, while minor carbonate signals are consistent with the observed calcite veins and the elevated CaO reported in the chemical analysis. These assignments fall within the documented absorption ranges for feldspars, calcite, quartz, and phyllosilicates reported in previous studies^85^. The ophiolitic schist (Fig. 9b) displays a very broad OH band at 3416 cm^− 1^ together with an absorption at 1631 cm^− 1^, reflecting the abundance of phyllosilicates including muscovite, chlorite, and talc. Peaks at 1385, 1119, and 1077 cm^− 1^ correspond to C–H and C–O vibrations, while the strong absorption at 874 cm^− 1^ is characteristic of calcite, in agreement with the modal dominance of calcite and talc and the relatively high Al_2_O_3_ and alkali contents measured chemically. This assignment is reinforced by published carbonate standards, which place calcite absorptions near 872 cm^− 1^ and 1395 cm^− 1^^86^. The serpentinized peridotite (Fig. 9c) spectrum shows a pronounced OH band at 3365 cm^− 1^, typical of serpentine-group minerals, together with additional features at 2162 and 2022 cm^− 1^ that may be related to complex carbonate or sulfide phases. A sharp band at 1507 cm^− 1^, together with multiple peaks in the fingerprint region such as 952, 754, and 648 cm^− 1^, further confirm the coexistence of serpentine, talc, carbonates, and oxides, which agrees with the ultramafic chemistry marked by high MgO and CaO, low SiO_2_, and elevated SO_3_ contents.

In contrast, the hornblende gabbro-diorite spectrum (Fig. 9d) is dominated by Si–O framework absorptions near 989, 873, and 712 cm^− 1^, reflecting its plagioclase and hornblende composition.

The OH band at 3385 cm^− 1^ is present but relatively weak, which is consistent with the petrographic description of limited secondary alteration and with its chemical profile showing moderate Al_2_O_3_, CaO, and FeO but comparatively low LOI. From results, it’s safe to conclude that the FTIR spectra align closely with the mineralogical and chemical characteristics of each rock type: spectra rich in OH and carbonate features correspond to samples with abundant hydrous silicates and carbonates, while spectra dominated by Si–O vibrations and subdued OH bands are typical of more crystalline, less altered lithologies such as the gabbro-diorite.

### Radiation shielding results

**Fig. 10:**
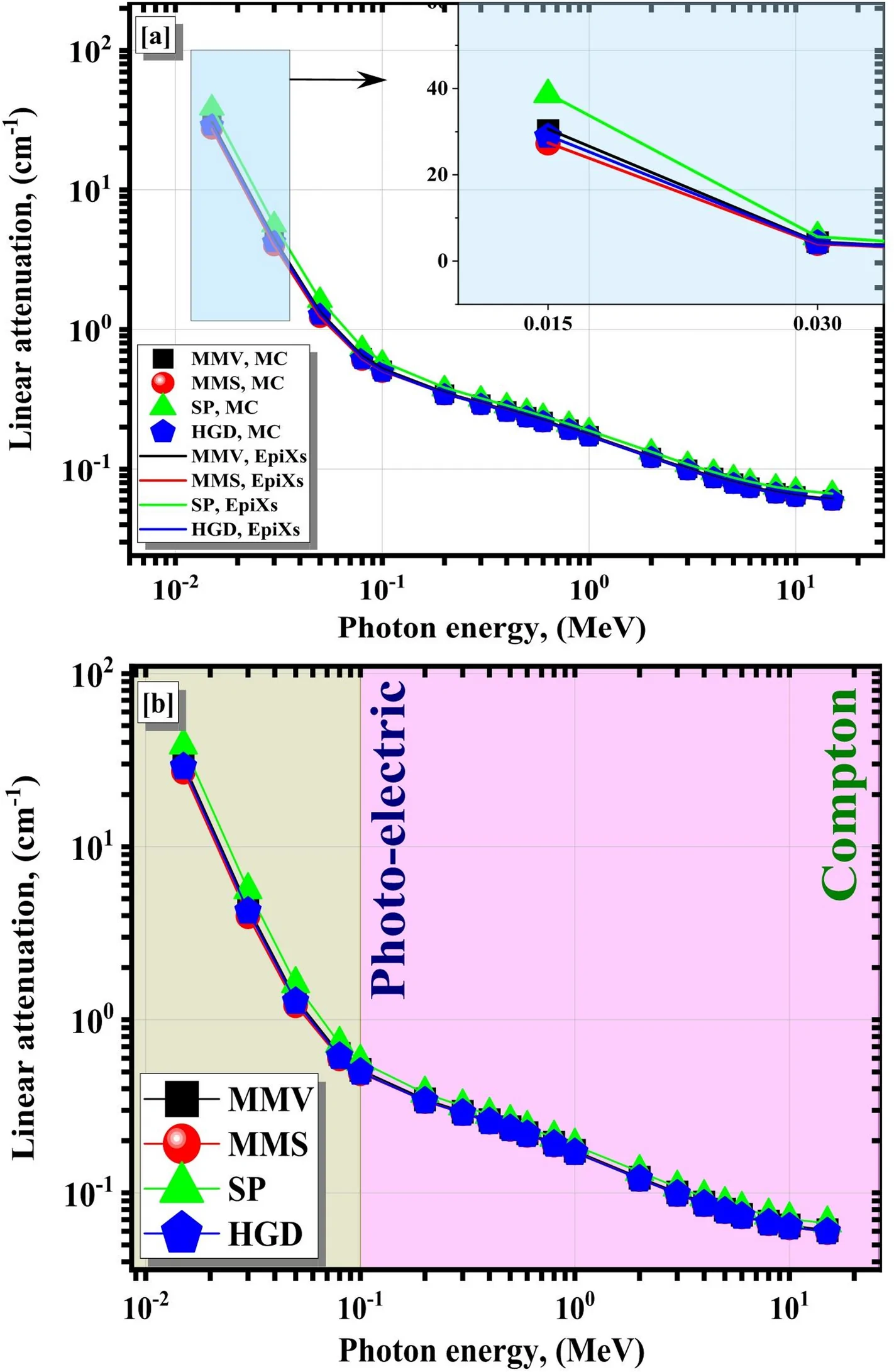
The γ-linear attenuation (a) calculated from MC and EpiXs, and (b) of the two regions of γ-interaction with the collected ophiolitic rocks.

The radiation shielding efficiency of the investigated samples was evaluated across a wide energy spectrum (0.015 to 15 MeV). The µ values were calculated using MC simulations and validated via the EpiXs software. Additionally, experimental measurements were conducted at three distinct energy lines (0.661, 1.173, and 1.332 MeV) to ensure accuracy. The results demonstrate that µvalues decreased significantly as energy increased up to 15 MeV, dropping from 30.422 to 0.061 cm^− 1^ for MMV, 27.232 to 0.060 cm^− 1^ for MMS, 38.556 to 0.067 cm^− 1^ for SP, and 28.915 to 0.060 cm^− 1^ for HGD. A pronounced non-linear decline in µ is observed in the low-energy region (Eγ$\:\le\:$0.200 MeV) for the ophiolitic rock samples, as introduced in Fig. 10b. This behavior is primarily attributed to the dominance of the Photoelectric Effect (PE), where the interaction cross-section is inversely proportional to the photon energy^87^. Within this low-energy range, an exponential reduction in µ was recorded, falling from 30.422 to 0.347 cm^− 1^ for MMV, and similarly for other samples. Finally, the comparison between MC simulations and EpiXs results revealed strong agreement, with a mean relative deviation (φ) of only 2.431%, as cleared in Fig. 10a.

In the intermediate energy region (E ≤ 0.200 MeV), Compton scattering (COM) becomes the predominant interaction mechanism, leading to a more gradual decrease in the µ values (Fig. 10b)^88^. The interaction cross-section for Compton scattering is approximately inversely proportional to the incident photon energy, which explains the sustained reduction in attenuation efficiency as energy increases^89^. Unlike the photoelectric effect, which drops sharply, the COM interaction remains significant over a broader energy range. Consequently, within the 0.300 to 15 MeV range, a progressive decline in µ was observed for all samples. Specifically, µ values decreased from 0.295 to 0.061 cm^− 1^ for MMV, 0.289 to 0.060 cm^− 1^ for MMS, 0.317 to 0.067 cm^− 1^ for SP, and 0.288 to 0.060 cm^− 1^ for the HGD sample. This trend confirms that as photon energy increases, the probability of photon-matter interaction decreases, allowing for greater penetration through the ophiolitic rock shields.

**Fig. 11:**
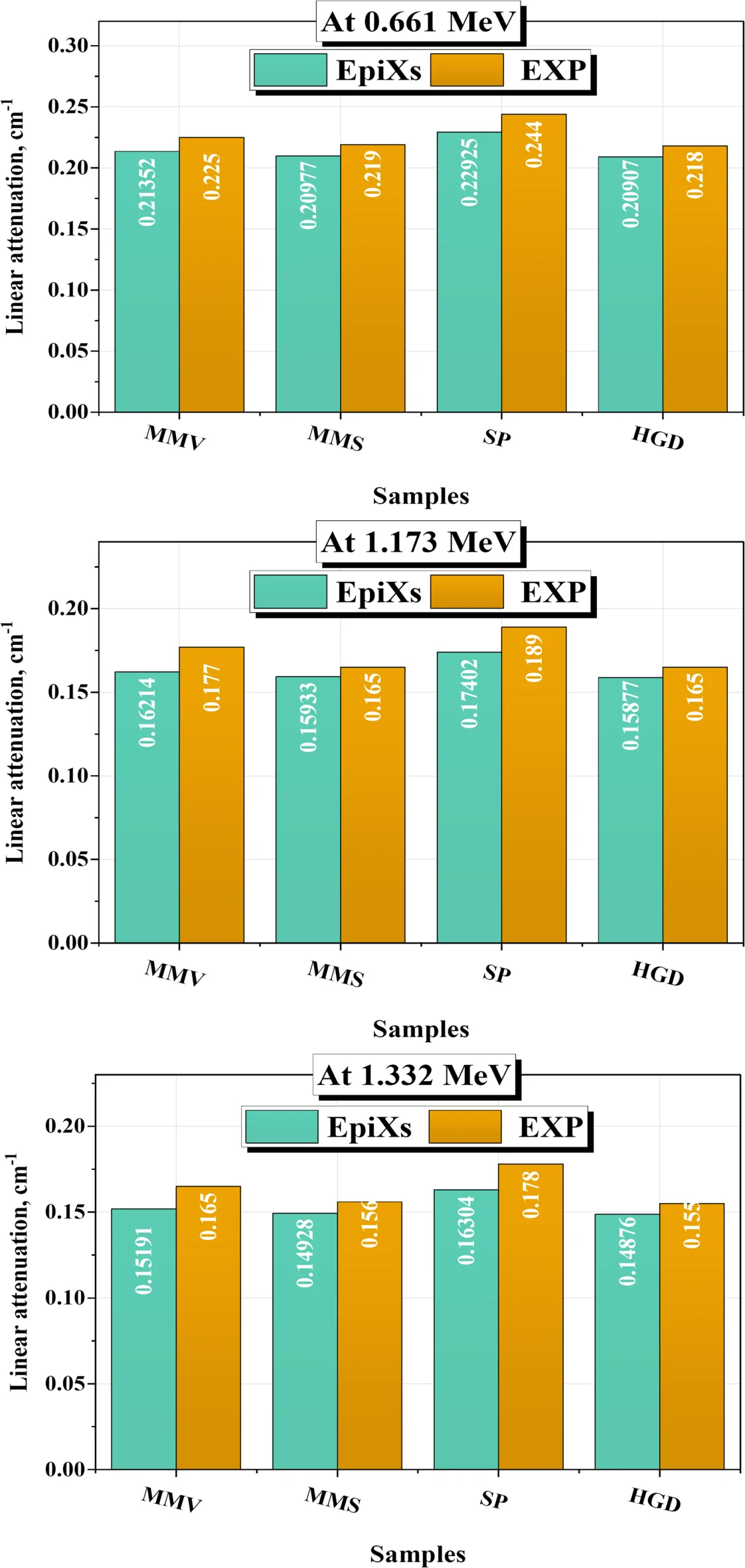
The experimental/theoretical linear attenuation of the collected ophiolitic rocks.

As illustrated in Fig. 11(a-c), the experimental measurements conducted at photon energies of 0.661, 1.173, and 1.332 MeV were utilized to validate the calculated µ values. A comparison reveals that the experimentally measured µ values for the prepared composites are consistently higher than the computed results. The relative discrepancy between the two approaches ranged from − 3.557% to -9.175%. The overall experimental uncertainty associated with the gamma-ray attenuation measurements was estimated to be less than 8%, considering counting statistics, sample thickness variation, density measurements, and geometrical alignment uncertainties. The observed discrepancies between the experimental and simulated attenuation coefficients can be attributed to several factors inherent to both the measurement procedure and the numerical model. Although considerable care was taken during sample preparation, natural ophiolitic rocks are intrinsically heterogeneous materials characterized by mineralogical, textural, and compositional variations at different spatial scales. Consequently, local fluctuations in density and elemental distribution may exist within individual specimens, whereas the MC simulations assume a homogeneous medium based on bulk chemical compositions and average density values. In addition, small uncertainties in specimen thickness measurements can influence the calculated attenuation coefficients because the Beer–Lambert attenuation relationship is highly sensitive to photon path length. Detector-related factors, including counting statistics, background subtraction, peak fitting procedures, and finite counting times associated with the HPGe system, may also contribute to minor variations in the experimentally determined photon intensities. Furthermore, the MC model represents an idealized geometry, while slight deviations in the actual experimental setup, such as source-to-sample positioning, detector alignment, collimator geometry, and contributions from scattered radiation originating from surrounding materials, cannot be perfectly replicated. Therefore, the combined influence of material heterogeneity, dimensional tolerances, counting statistical uncertainties, and unavoidable simplifications in the simulation geometry is considered the most probable explanation for the observed deviations. Nevertheless, the relatively small discrepancy range and the strong agreement between the experimental, MC, and EpiXs results confirm the reliability and robustness of the adopted methodology.

**Fig. 12:**
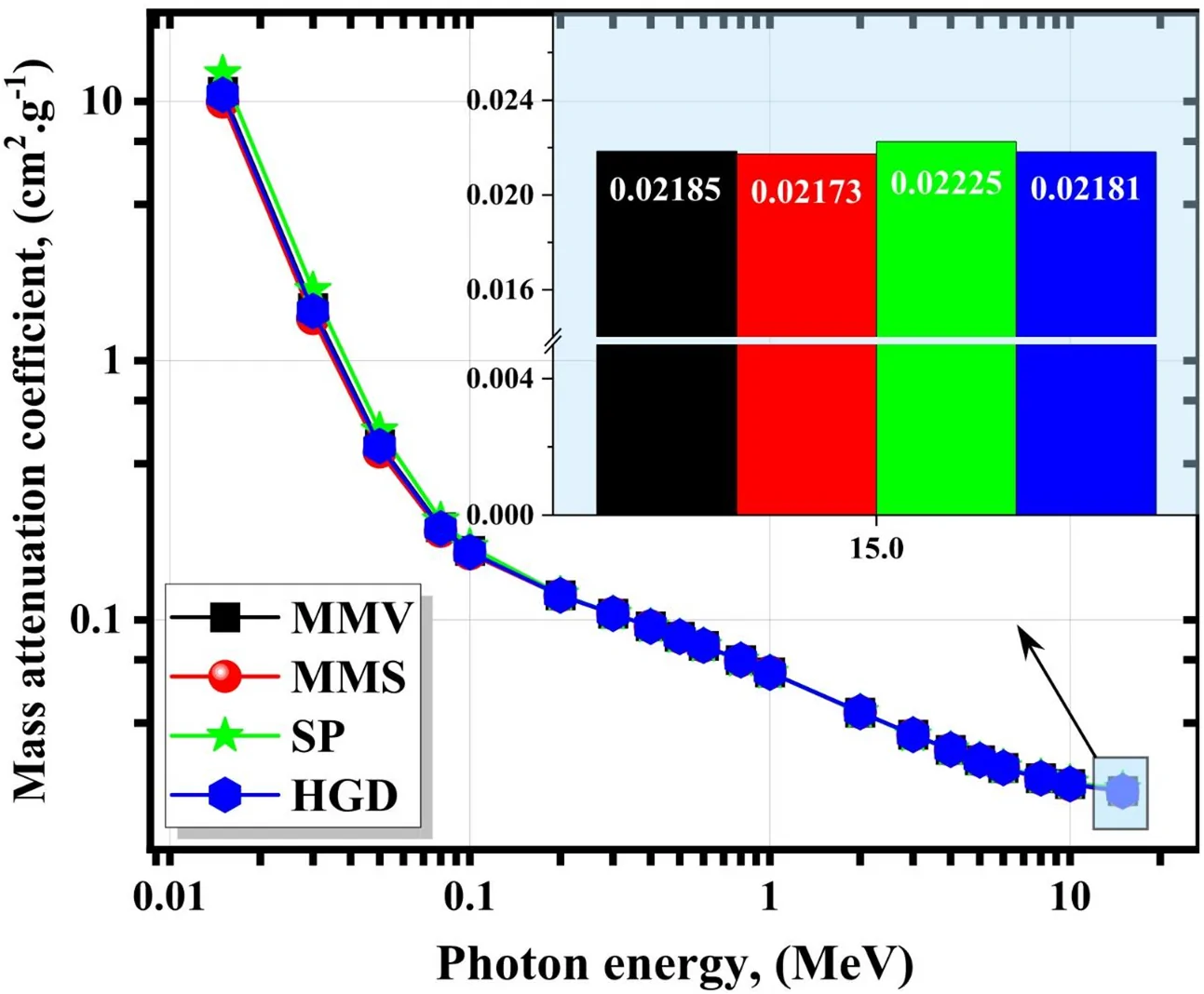
The obtained MAC vs. the photon energy for the collected ophiolitic rocks.

The mass attenuation coefficient values followed a similar trend to that of the linear attenuation coefficient. For the MMV sample, MAC values decreased from 10.904 to 0.022 cm^2^.g^− 1^. Similarly, the values dropped from 9.939 to 0.022 cm^2^.g^− 1^ for MMS, from 12.852 to 0.022 cm^2^.g^− 1^ for SP, and from 10.591 to 0.022 cm^2^.g^− 1^ for the HGD sample, as shown in Fig. 12. This consistency between µ and MAC trends underscores the reliability of the attenuation data across the investigated energy range.

**Fig. 13:**
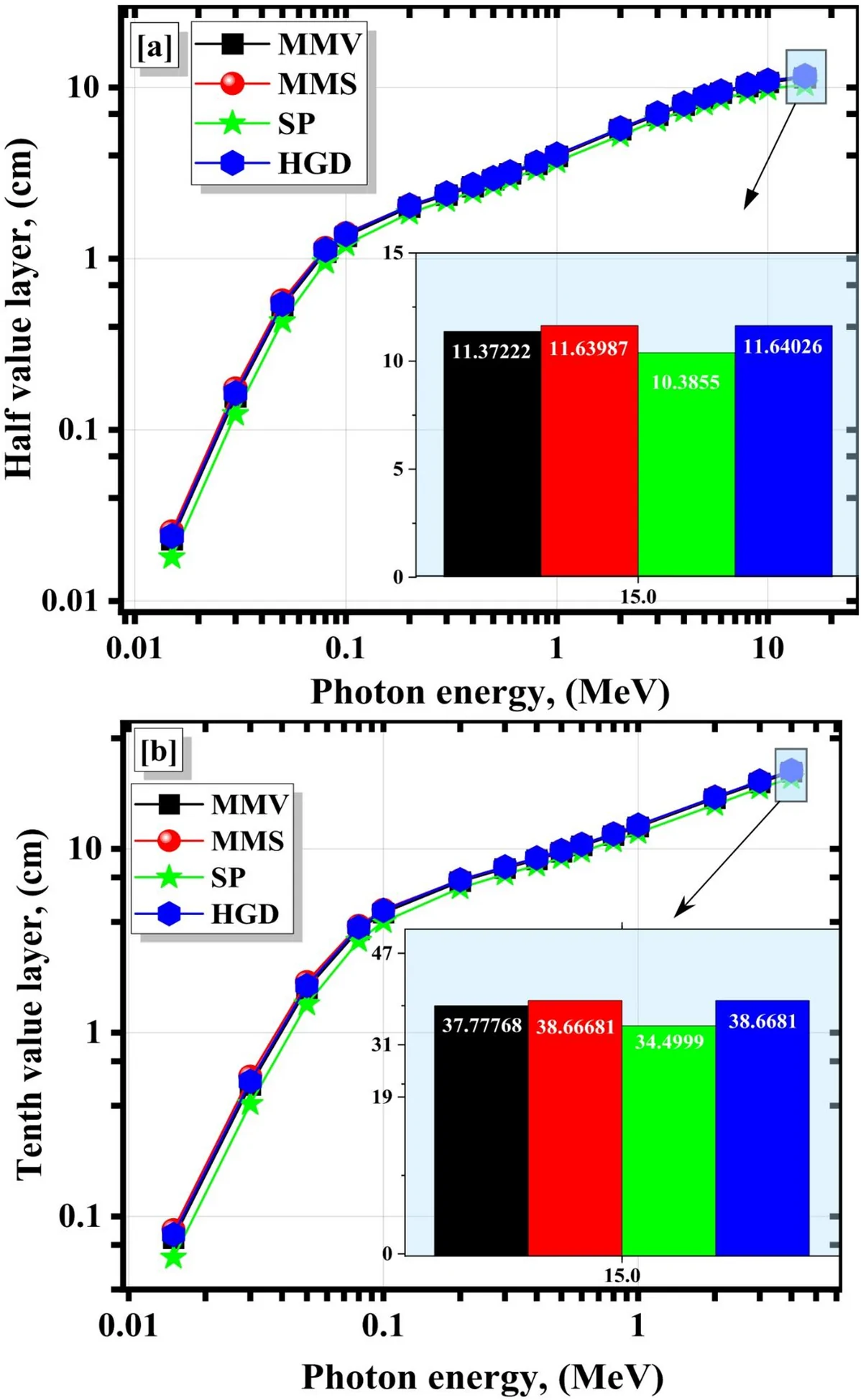
(a) The HVL, and (b) the TVL of the collected ophiolitic rocks*. * Derived parameter uncertainties were calculated from µ uncertainties using error propagation.

In the evaluation of shielding effectiveness, lower magnitudes for both the HVL and TVL are indicative of superior radiation protection performance at a specified energy. This is because a lower HVL implies that the radiation intensity is halved within a significantly smaller spatial region, suggesting a high macroscopic interaction cross-section^89^. As seen in Fig. 13a, the HVL for the investigated samples increased significantly across the energy spectrum (up to 15 MeV), ranging from 0.023 to 11.372 cm for MMV, 0.025 to 11.640 cm for MMS, 0.018 to 10.386 cm for SP, and 0.024 to 11.640 cm for HGD. The TVL, shown in Fig. 13b, followed an identical trend, expanding from 0.076 to 37.778 cm (MMV), 0.085 to 38.667 cm (MMS), 0.060 to 34.500 cm (SP), and 0.080 to 38.668 cm (HGD). These findings underscore that the SP sample possesses the most efficient attenuation profile, characterized by the highest µ values and the lowest HVL and TVL requirements. This superior performance is directly attributed to its high bulk density (3.00 g/cm^3^) and the elevated concentrations of heavy metallic oxides, specifically FeO (13.24%), MgO (18.47%), and TiO_2_ (1.902%). Consequently, the samples can be ranked in descending order of shielding effectiveness as: SP > MMV > HGD > MMS.

**Fig. 14:**
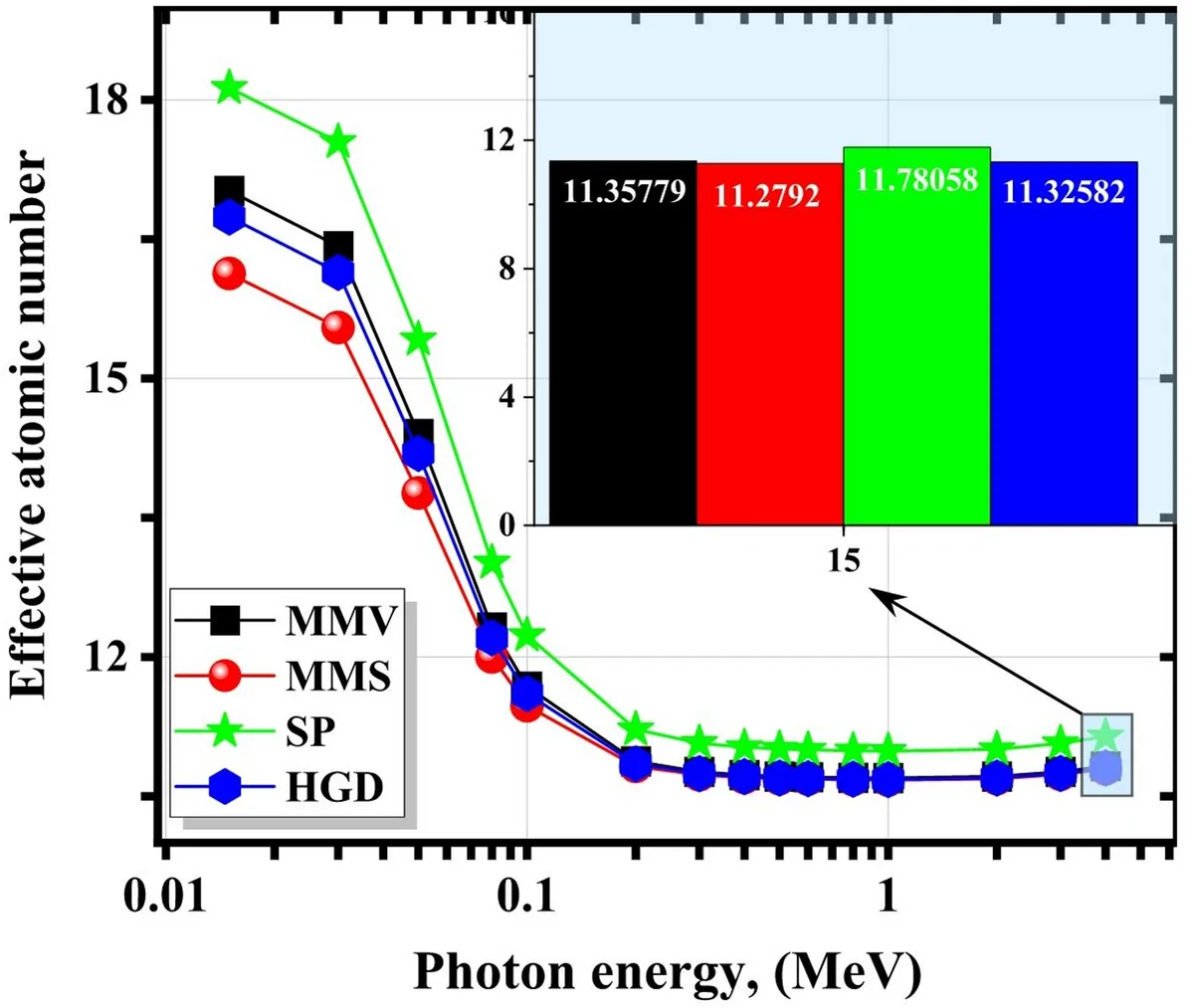
The Z_eff_ for the collected ophiolitic rocks vs. photon energy.

The energy dependence of Z_eff_ is primarily governed by the dominant photon interaction mechanism within each energy region. At low photon energies, the photoelectric effect predominates, resulting in higher Z_eff_ values due to the strong dependence of the interaction cross-section on atomic number. As photon energy increases and Compton scattering becomes dominant, the variation in Z_eff_ becomes less pronounced. Figure 14 illustrates the energy-dependent fluctuations of Z_eff_ for the investigated ophiolitic compounds. Generally, an elevated Z_eff_ correlates with an enhanced cross-section for photon interactions, thereby improving the material’s shielding efficacy^90^. In this study, the Z_eff_ values exhibited a distinct downward trend across the analyzed energy range: decreasing from 17.021 to 11.358 for MMV, 16.129 to 11.279 for MMS, 18.128 to 11.781 for SP, and 16.739 to 11.326 for the HGD sample. The higher Z_eff_ values observed in the low-energy region are primarily governed by the PE, where the interaction probability is proportional to Z^4^-Z^5^. Consequently, the SP sample, possessing the highest Z_eff_ profile, demonstrates a superior capacity for attenuating high-energy gamma radiation compared to the other ophiolitic specimens^91^.

**Fig. 15:**
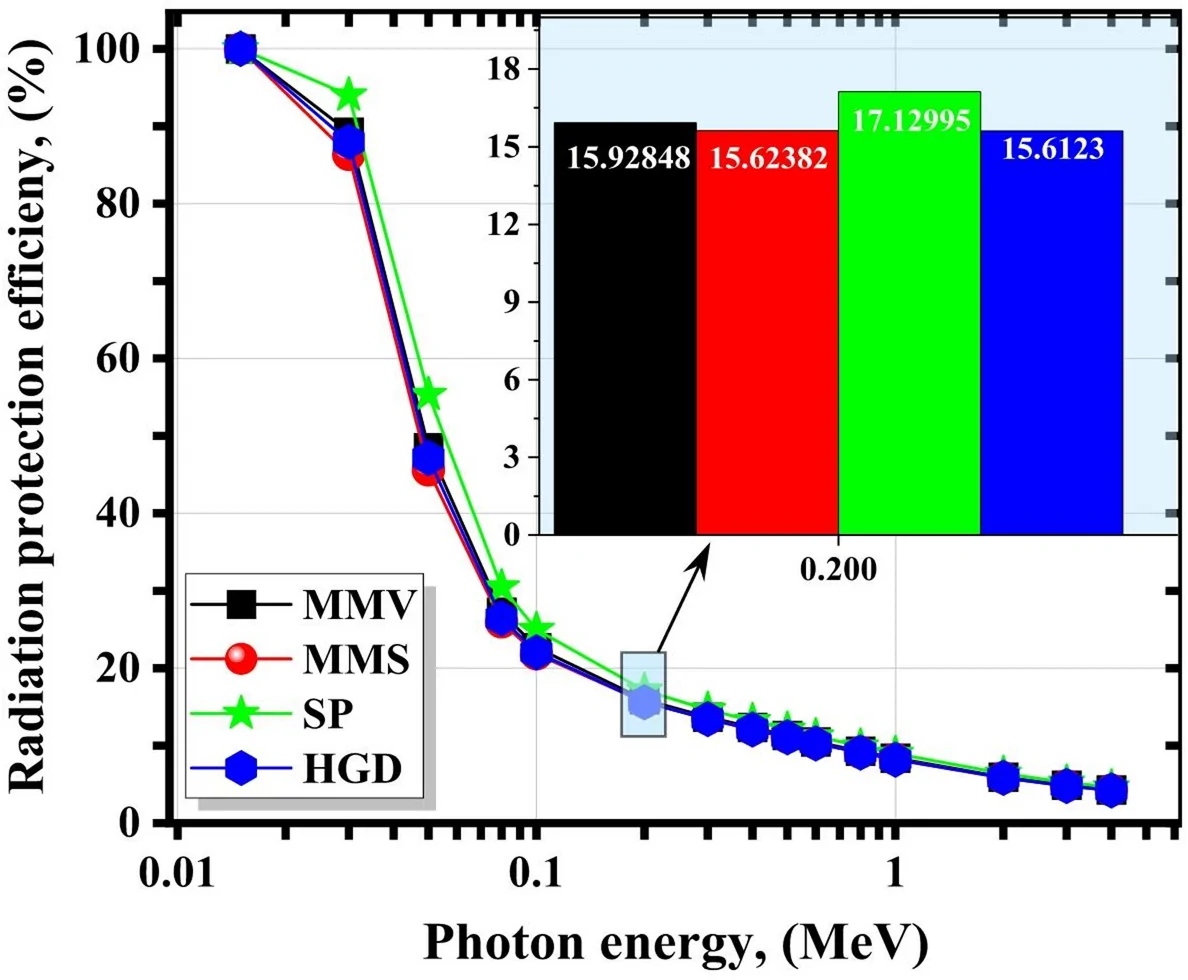
The RP_eff_ for the collected ophiolitic rocks vs. photon energy.

The findings of this study indicate that the radiation attenuation capacity of the investigated materials is highly dependent on the incident γ-ray energy (Eγ). Achieving robust radiation shielding across both low and high energy spectra (0.015–15 MeV) is a primary requirement for shielding applications. The reduction in radiation protection efficiency (RP_eff_) with increasing photon energy is attributed to the transition from the photoelectric absorption region to the compton scattering region, where photon interaction probabilities become lower and gamma-ray penetration through the shielding material increases. The RP_eff_ values for the ophiolitic rock samples are depicted in Fig. 15. At the lowest energy level of 0.015 MeV, all samples (MMV, MMS, SP, and HGD) exhibited a near-total attenuation efficiency of ~ 100%, primarily due to the dominance of the Photoelectric Effect. However, as Eγ increases, the probability of photon-electron interactions within the ophiolitic matrix diminishes. This leads to a higher population of scattered photons, which negatively impacts the RP_eff_. For instance, as energy levels reached 0.200 MeV, a sharp decline in shielding efficiency was observed, with RPE values dropping to 15.928% (MMV), 15.624% (MMS), 17.130% (SP), and 15.612% (HGD). This trend underscores the transition from complete absorption in the low-energy regime to the Compton Scattering region, where the material’s transparency to ionizing radiation increases significantly. RP_eff_ uncertainties originated from the same MC tally statistics used to derive the attenuation coefficients.

Also, in order to assess the neutron shielding capabilities of the investigated mafic and ultramafic rock samples, the fast neutron removal cross section (FCS), half-value layer (HVL), and relaxation length (λ) were evaluated (Table 2). These parameters are among the most widely accepted indicators for assessing the effectiveness of materials against fast neutrons. The fast neutron removal cross section represents the probability of removing fast neutrons from an incident neutron beam through scattering and absorption interactions and is therefore considered a direct measure of neutron attenuation efficiency. Materials exhibiting higher FCS values are generally more effective neutron shields because they provide a greater likelihood of neutron interaction per unit thickness. The HVL defines the thickness of material required to reduce the incident neutron intensity by 50%, making it a practical engineering parameter for shield design and optimization^26^. Lower HVL values indicate superior shielding performance and reduced material requirements. Similarly, λ values describes the characteristic distance over which the neutron flux decreases exponentially within a shielding medium and provides insight into the attenuation behavior of neutrons as they penetrate the material. A shorter relaxation length corresponds to a more rapid reduction in neutron flux and consequently a higher shielding efficiency. Since neutron interactions are strongly influenced by elemental composition, density, and the presence of light elements capable of moderating neutrons as well as heavier elements that contribute to neutron capture, these parameters collectively provide a comprehensive evaluation of the neutron shielding potential of geological materials.

Results showed that the FCS values that were measured ranged from 0.090 to 0.097 cm^− 1^. The serpentinized peridotite (SP) material has the highest FCS value (0.097 cm^− 1^), which indicates that it has the greatest potential to remove the fast neutrons from the incident neutron flux. This is the case among the materials that were studied. The superior performance can be attributed to its unique mineral composition, particularly the elevated levels of magnesium oxide (18.47 weight%) and iron oxide (13.24 weight%), as well as the highest density of all samples (3.00 g per cubic centimeter). Both the neutron interaction processes and the chance of neutron attenuation within the material are enhanced by these characteristics, which are advantageous for the neutron interaction processes. The hornblende gabbro-diorite (HGD) sample had the lowest FCS value (0.090 cm^− 1^), followed by the mafic metasediment (MMS) and mafic metavolcanic (MMV) samples. The FCS value was the lowest for the HGD sample. The differences are not particularly significant; nonetheless, they do demonstrate that chemical composition and density have an effect on the effectiveness of neutron shielding. It was found that the HVL readings varied from 7.121 cm to 7.667 cm. The SP sample demonstrated the highest performance in neutron attenuation once more with a minimal HVL of 7.121 cm. This is due to the fact that a lower value of HVL indicates higher shielding capabilities. This indicates that 7.121 cm of SP is sufficient to bring about a 50% reduction in the strength of the fast neutron. When compared to the other samples, the HGD sample exhibited the highest HVL (7.667 cm), which indicates that the shielding efficacy was somewhat lower and that a more substantial layer was required to achieve the same level of attenuation. When it came to relaxation length, it was discovered that the values varied between 10.273 and 11.061 centimeters and followed a similar pattern. The SP sample was found to have the shortest relaxation length, which was measured at 10.273 centimeters. This indicates that the level of neutron flux within the material decreased more rapidly. HGD, on the other hand, has the longest relaxation length (11.061 cm), which implies that the attenuation rate is relatively lower and, as a result, the neutron shielding efficacy is lower.

The behavior that was observed provides evidence that the neutron shielding parameters are highly dependent on the element composition and bulk density. It is possible to explain the increased performance of the SP sample to its higher amount of magnesium and iron-bearing minerals, both of which are beneficial for neutron contact, as well as its relatively high density, which increases the number of interaction sites per unit volume. Based on the rocks that were examined, it can be concluded that SP is the most effective natural shielding material that may be utilized for the purpose of fast neutron attenuation.

In conclusion, the order of the neutron shielding performance according to FCS, HVL, and relaxation duration may be summarized as follows: SP > MMV ≈ MMS > HGD. This indicates that serpentinized peridotite is the most effective fast neutron shielding material among the geological materials that were tested.

**Table 2:** The FCS, HVL, and λ of the investigated rock samples.

Samples	FCS, cm^− 1^	HVL, cm	λ, cm
MMV	0.092	7.514	10.840
MMS	0.091	7.632	11.010
SP	0.097	7.121	10.273
HGD	0.090	7.667	11.061

## Conclusions

Ophiolitic rocks from the Eastern Desert of Egypt, comprising serpentinized peridotites, hornblende gabbro-diorites, mafic metavolcanics, and ophiolitic schists, are evaluated as sustainable and cost-effective alternatives to conventional synthetic shielding materials. These lithologies are considered due to their distinctive mechanical, mineralogical, and geochemical attributes, which collectively position them as promising materials for radiation shielding applications. Their elevated bulk densities, primarily resulting from enrichment in Fe–Mg–bearing minerals, significantly enhance their attenuation capacity relative to common granitoids and sedimentary rocks. Petrographic observations and XRD analyses confirm the presence of mineral assemblages favorable for radiation absorption, including serpentine, olivine, pyroxene, hornblende, talc, and magnetite. These phases contribute synergistically to both high density and improved attenuation efficiency. Furthermore, geochemical data reveal notable compositional variations among the examined rock types, with consistently low silica and elevated MgO and FeO contents, which further augment their potential as natural, sustainable, and economically viable shielding materials. This research addresses a critical gap in the literature by providing the first comprehensive multi-analytical assessment (XRD, TGA, FTIR, experimental γ-ray measurements, and Monte Carlo simulations) of Egyptian ophiolitic rocks for radiation shielding applications. The abundance and widespread distribution of these ophiolitic terranes throughout the Arabian-Nubian Shield presents unprecedented opportunities for large-scale utilization of these naturally occurring materials in nuclear facilities, medical institutions, and construction industries requiring radiation protection. The integration of advanced analytical techniques with both experimental and computational validation establishes a robust framework for evaluating natural geological materials as radiation shields, potentially revolutionizing the approach to sustainable radiation protection strategies. The conclusions are drawn as follows:

- Geochemical analysis established compositional ranges from ultramafic (31.08 wt% SiO_2_, 18.47 wt% MgO) to intermediate compositions (55.96 wt% SiO_2_), with serpentinized peridotites showing the most distinctive ultramafic signature.
- XRD analysis revealed distinct mineralogical assemblages for each rock type: serpentine-dominated ultramafic compositions in peridotites, phyllosilicate-rich assemblages in ophiolitic schists, plagioclase–actinolite–chlorite dominated assemblage typical for greenschist facies in mafic metavolcanics, and plagioclase–hornblende–quartz–biotite assemblages in metagabbro-diorites.
- TGA analysis demonstrated variable thermal behavior with total weight losses ranging from 6.6% (metagabbro-diorite) to 16% (mafic metavolcanics).
- The relative thermal stability of the investigated rock types can be hierarchically arranged as follows: metagabbro-diorite > Ophiolitic schist (7%) > Serpentinized peridotites (9%) > Mafic metavolcanics (16%).
- Weight loss patterns correlate with the abundance of hydrous minerals, carbonates, and alteration phases, providing crucial data for high-temperature applications.
- FTIR spectroscopy confirmed the presence of hydroxyl-bearing phases, carbonate groups, and silicate framework vibrations that correlate directly with XRD and geochemical data.
- Serpentinized peridotites demonstrated the highest radiation attenuation efficiency with linear attenuation coefficients ranging from 38.556 to 0.067 cm^-1^ across the 0.015-15 MeV energy spectrum.
- The radiation protection effectiveness reached nearly 100% at low energies (0.015 MeV) and maintained significant attenuation (17.130%) even at higher energies (0.200 MeV).
- Experimental validation using HPGe detector confirmed theoretical predictions with acceptable deviation (-3.557% to -9.175%).
- The radiation shielding effectiveness follows the order: Serpentinized peridotites > Mafic metavolcanics > metagabbro-diorite > Ophiolitic schist. This ranking directly correlates with bulk density (3.00 > 2.79 > 2.73 > 2.74 g/cm³), heavy element content (FeO, MgO, TiO_2_), and specific mineral assemblages.
- Effective atomic number values ranged from 11.279 to 18.128, with serpentinized peridotites showing the highest values across all energy ranges.
- Neutron shielding assessment demonstrated that serpentinized peridotites possess the highest fast neutron removal cross-section  (0.097 cm⁻¹) and the lowest HVL (7.121 cm) and relaxation length (10.273 cm), confirming their superior capability for fast-neutron attenuation and highlighting their suitability for dual gamma–neutron shielding applications.
- The order of the neutron shielding performance according to FCS, HVL, and relaxation duration may be summarized as follows: SP > MMV ≈ MMS > HGD.

This study establishes a strong foundation for the development of natural ophiolitic rocks as sustainable radiation shielding materials, opening new avenues for both fundamental research and practical applications in radiation protection technology. Future research should encompass optimization of industrial-scale processing techniques, development of concrete composites incorporating ophiolitic aggregates and cement replacement powder, and long-term radiation-induced changes in mineralogical properties require comprehensive evaluation. Advanced characterization including neutron activation analysis, microstructural studies, and complex Monte Carlo simulations will enhance understanding of radiation-material interactions. Long-term performance studies under accelerated aging conditions, comprehensive life cycle assessments, and integration with green building standards are essential for practical implementation. Additionally, investigating mechanical properties, structural engineering applications, and cost-benefit analyses compared to conventional materials will facilitate widespread adoption of these sustainable radiation shielding solutions.

## Data Availability

All data generated or analyzed during this study are included in this published article.
